# PRL-mediated STAT5B/ARRB2 pathway promotes the progression of prostate cancer through the activation of MAPK signaling

**DOI:** 10.1038/s41419-023-06362-2

**Published:** 2024-02-10

**Authors:** Tao Yang, Yongnan Chi, Xin’an Wang, Chengdang Xu, Xi Chen, Ying Liu, Shengsong Huang, Xuyou Zhu, Haoyang Zhang, Hui Zhuo, Denglong Wu

**Affiliations:** 1 Department of Urology, Tongji Hospital, School of Medicine, Tongji University, Shanghai, China; 2 Department of Urology, The Third People’s Hospital of Chengdu/The Affiliated Hospital of Southwest Jiaotong University, Chengdu, Sichuan China; 3 Department of Pathology, Tongji Hospital, School of Medicine, Tongji University, Shanghai, China; 4 Department of Pathology, Baoshan Branch, Shuguang Hospital, Shanghai University of Traditional Chinese Medicine, Shanghai, China

**Keywords:** Pathogenesis, Prostate cancer

## Abstract

Previous study showed that higher expression of prolactin (PRL) was found in CRPC samples compared with hormone-naive prostate cancer (HNPC) and benign prostatic hyperplasia (BPH) samples. We further investigate the function of PRL in prostate cancer (PCa) and explored its downstream effects. We found heterogeneous expression of the PRLR in clinical prostate samples. The VCaP and 22Rv1 cells exhibited PRLR expression. Among the downstream proteins, STAT5B was the dominant subtype in clinical samples and cell lines. Human recombinant PRL stimulation of PCa cells with PRLR expression resulted in increased phosphorylation of STAT5B(pSTAT5B) and progression of PCa in vitro and in vivo, and STAT5B knockdown can suppress the malignant behavior of PCa. To understand the mechanism further, we performed Bioinformatic analysis, ChIP qPCR, and luciferase reporter gene assay. The results revealed that ARRB2 was the transcription target gene of STAT5B, and higher expression of ARRB2 was related to higher aggression and poorer prognosis of PCa. Additionally, Gene set enrichment analysis indicated that higher expression of ARRB2 was significantly enriched in the MAPK signaling pathway. Immunohistochemistry (IHC) demonstrated elevated pSTAT5B, ARRB2, and pERK1/2 expression levels in CRPC tissues compared to HNPC and BPH. Mechanically, ARRB2 enhanced the activation of the MAPK pathway by binding to ERK1/2, thereby promoting the phosphorylation of ERK1/2 (pERK1/2). In conclusion, our study demonstrated that PRL stimulation can promote the progression of PCa through STAT5B/ARRB2 pathway and activation of MAPK signaling, which can be suppressed by intervention targeting STAT5B. Blockade of the STAT5B can be a potential therapeutic target for PCa.

## Introduction

Metastatic castration-resistant prostate cancer (mCRPC) is an advanced disease that develops in many patients after endocrine therapy [1]. Resistance to traditional and next-generation hormone therapy drugs, such as abiraterone acetate (AA) or enzalutamide, is common in managing metastatic prostate cancer [2, 3]. Once patients become resistant to these treatments, the disease progresses rapidly and can be fatal. The mechanism of castration-resistant progression of prostate cancer (PCa) has been extensively researched. The androgen receptor-related pathway has been identified as a pivotal role in prostate cancer [2, 3]. Resistance to different stages of androgen blockers leads to increased malignancy and heterogeneity of prostate cancer [4, 5], such as the trans-differentiation of classical prostate carcinoma to neuroendocrine prostate cancer (NEPC) [6, 7] and AR-negative prostate cancer [8]. Studies have revealed that the abnormal activation of other signaling pathways is also critical for prostate cancer progression. For example, the Wnt/β-catenin signaling has been shown to contribute to prostate cancer cell proliferation, differentiation, and epithelial-mesenchymal transition [9–11]. Additionally, the AKT/mTOR pathway promotes the growth of androgen-independent prostate tumors [12, 13]. Therefore, understanding the independent and mutual functions of these pathways in the progression of prostate cancer, particularly for prostate cancer resistance to different hormonal therapy drugs, is crucial.

The role of prolactin (PRL) in prostate cancer had been investigated in some previous studies. Related study had found that the PRL and PRL receptor (PRLR) was locally expressed in prostate epithelium [14], and a significantly higher serum prolactin were found in patients with metastatic disease, compared with the patients without metastases [15]. Nevalainen MT. et al had found that local prolactin protein expression and constitutive activation of STAT5 are associated with high histologic grade of clinical prostate cancer [16, 17]. However, no antitumor activity was found in the clinical trial of PRLR antagonist LFA102 in patients with mCRPC and advanced breast cancer [18, 19]. In summary, the expression and functions of the down streaming signals of PRL in prostate cancer was not figured out thoroughly, and the role of PRL in mCRPC stage is unclear.

In our previous study, we analyzed the prognostic value of the baseline serum hormones in patients with metastatic castration-resistant prostate cancer (mCRPC) treated with abiraterone, we found that higher serum PRL was a poor prognostic factor for patients with mCRPC, and higher expression of PRL was found in CRPC tissues compared to hormone naive prostate cancer (HNPC) and begin prostate hyperplasia (BPH) tissues. Additionally, PRL expression was detected in bone metastasis of patients with mCRPC, but not detected in normal bone tissue, and a positive correlation was found between serum PRL levels and bone metastasis volume [20]. Based on these results, we further investigated the role of PRL and its downstream signaling pathways in the progression of PCa, we found that a heterogeneous expression of the PRLR in clinical prostate samples and PCa cell lines, and the STAT5B was the major subtype in the down streaming signaling of PRL in PCa. Mechanically, we found that STAT5B regulated the expression of ARRB2 and promotes PCa progression by activating MAPK signaling. In conclusion, our study indicated that STAT5B can be a potential therapeutic target for PCa.

## Results

### Downstream signaling of PRL in prostate cancer

**Fig. 1:**
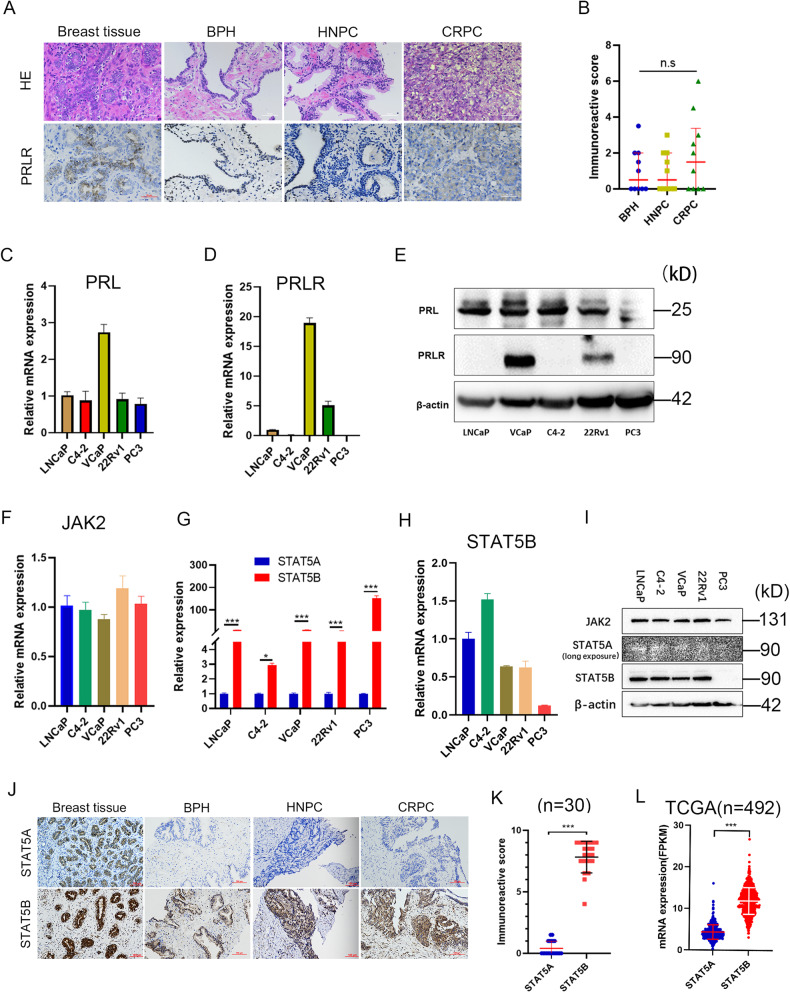
Expression of downstream molecules of prolactin (PRL) in prostate cancer tissues and cell lines. **A** Immunohistochemical (IHC) staining of prolactin receptor (PRLR) in prostate tissues including castration resistant prostate cancer(CRPC), hormone naive prostate cancer (HNPC) and benign prostate hyperplasia (BPH). **B** Comparison of PRLR expression levels among different pathological tissues. **C**, **D** mRNA expression levels of PRL and PRLR in different prostate cancer cell lines. **E** Protein levels of PRL and PRLR in different prostate cancer cell lines. **F** mRNA expression levels of JAK2 in different prostate cancer cell lines. **G** Comparison of mRNA expression levels between STAT5A and STAT5B in prostate cancer cell lines. **H** mRNA expression levels of STAT5B in different prostate cancer cell lines. **I** Protein levels of JAK2, STAT5A, and STAT5B in different prostate cancer cell lines. **J** IHC staining of STAT5A and STAT5B and (**K**) Comparison of STAT5A and STAT5B expression levels in prostate tissues. **L** Comparison of STAT5A and STAT5B expression levels in the The Cancer Genome Atlas (TCGA) database.

The binding of PRL to prolactin receptor (PRLR) initiates downstream signaling pathways and exerts the functions in human body [21]. Immunohistochemistry (IHC) analysis of clinical samples revealed a heterogenous expression of PRLR in PCa tissues (Fig. 1A), and no statistical significance existed between different pathological tissue types including CRPC (median IHC score: 1.5, 0.0–3.4), HNPC (0.5, 0.0–2.0), and BPH (0.5, 0.0–2.0) (Fig. 1B). The clinical features of patients with tissues for IHC are shown in Supplementary Table 1. We further investigated the expression levels of PRL and PRLR in PCa cell lines, including LNCaP, VCaP, C4–2, 22Rv1, and PC3, and the RT-qPCR and Western blotting (WB) revealed a universal expression of PRL in these cell lines. However, only VCaP and 22Rv1 cell lines showed expression of PRLR (Fig. 1C–E), and these two cell lines were used for further experiments therefore.

Related studies have validated that the Janus kinase 2 (JAK2) and signal transducer and activator of transcription 5 (STAT5) signaling is the pivotal downstream pathway of PRL [22]; STAT5 has two homologous proteins, STAT5A and STAT5B that are encoded by two individual genes [23], the phosphorylation STAT5(pSTAT5) is an active state after PRL stimulation [21], and the pSTAT5 can enter the nucleus and regulate target gene expression [24]. We further determined the expression of JAK2 and STAT5 in PCa cell lines, the downstream signaling molecules JAK2 and STAT5 were found to be expressed in PCa cell lines (Fig. 1F, I**)**, with STAT5B being the dominant subtype (Fig. 1G–I**)**. STAT5B (median IHC score: 8.0, 7.5–9.0) was also the predominant form observed in clinical samples compared with STAT5A (0.0, 0.0–1.0) (Fig. 1J, K), and the results were consistent with TCGA-PRAD database (Fig. 1L). These results showed that a heterogeneous expression of PRLR in PCa clinical specimens and cell lines, and STAT5B was the dominant protein type in the downstream signaling of PRL.

### Enhancement of prostate cancer malignancy through PRL-mediated STAT5B phosphorylation

**Fig. 2:**
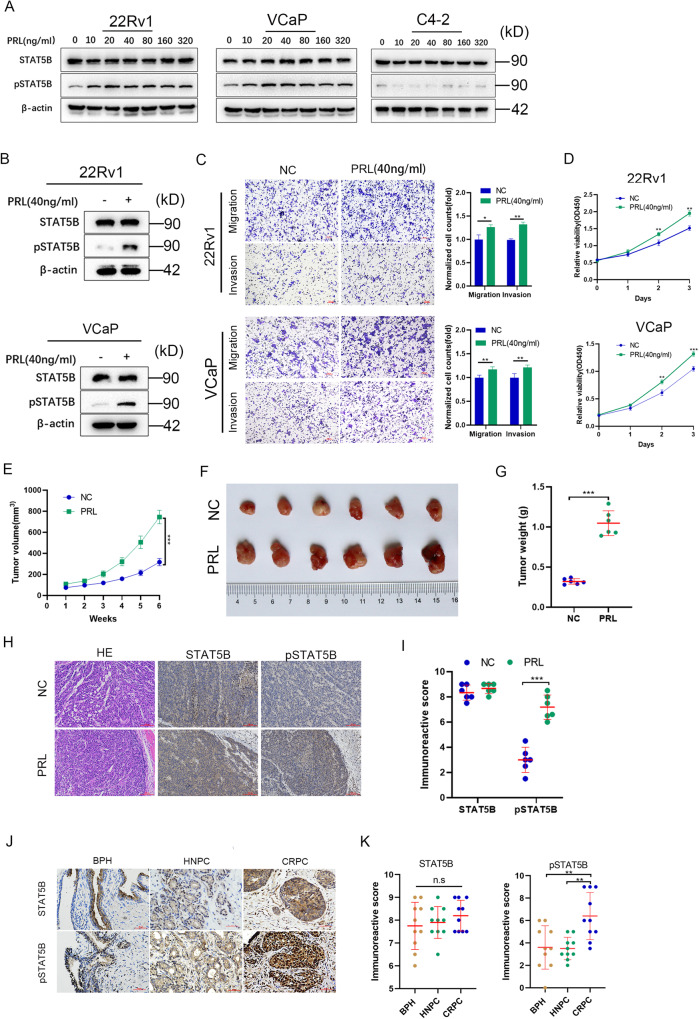
Prolactin (PRL) stimulation function on prostate cancer cell lines. **A** PRL stimulation experiment of PRLR positive (22Rv1 and VCaP) and negative (C4-2) cell lines and with different concentrations. **B** Influence of PRL stimulation on pSTAT5B levels in 22Rv1 and VCaP cells. **C** Effect of PRL stimulation on migration and invasion ability of 22Rv1 and VCaP cells. **D** Effect of PRL stimulation on proliferation ability of 22Rv1 and VCaP cells. **E**–**G** Role of PRL stimulation on xenografts growth in vivo. **H**, **I** Expression of STAT5B and pSTAT5B in xenografts was detected by immunohistochemical (IHC) staining. **J**, **K** Expression of STAT5B and pSTAT5B in different pathological prostate tissues including castration resistant prostate cancer(CRPC), hormone naive prostate cancer (HNPC) and benign prostate hyperplasia (BPH).

To further investigate the role of PRL in PCa, we conducted experiments to examine the effects of human recombinant PRL on different PCa cell lines. The focus was on 22Rv1 and VCaP cell lines with PRLR expression, and C4–2 cell lines without PRLR expression. The results showed that the increasing concentrations of PRL stimulation induced increased phosphorylation of STAT5B(pSTAT5B) in both 22Rv1 and VCaP cell lines, however, no affection was found in C4–2 cells (Fig. 2A). These results suggest that PRL activation enhanced STAT5B signaling in these cell lines. Interestingly, we observed that the levels of pSTAT5B reached a saturation point when the concentration of PRL reached 40 ng/ml. Furthermore, we investigated the influence of PRL stimulation on the malignant behaviors of PCa, and the results showed that PRL stimulation (40 ng/mL) could enhance the migration, invasion, and proliferation capacities of 22Rv1 and VCaP cells (Fig. 2B–D). To further investigate the function of PRL stimulation in vivo, Alzet minipumps containing human recombinant PRL or sterilized saline (control group) were implanted subcutaneously in mice with 22Rv1 xenograft, the results showed that PRL stimulation can significantly promoted tumor growth (Fig. 2E, F), and IHC staining showed the pSTAT5B levels was higher in PRL stimulation compared with control group (Fig. 2H, I**)**.The IHC of clinical samples also showed a higher level of pSTAT5B in CRPC tissues(median IHC score: 6.0, 4.8–9.0) compared with HNPC(3.3, 2.9–4.3) and BPH(3.5, 2.0–5.2) tissues (Fig. 2J, K). Overall, these results demonstrated that PRL stimulation can enhance the malignancy of PCa, and the pSTAT5B was the key effector molecular of PRL.

### Inhibition of STAT5B blocked the effects of PRL stimulation on prostate cancer

**Fig. 3:**
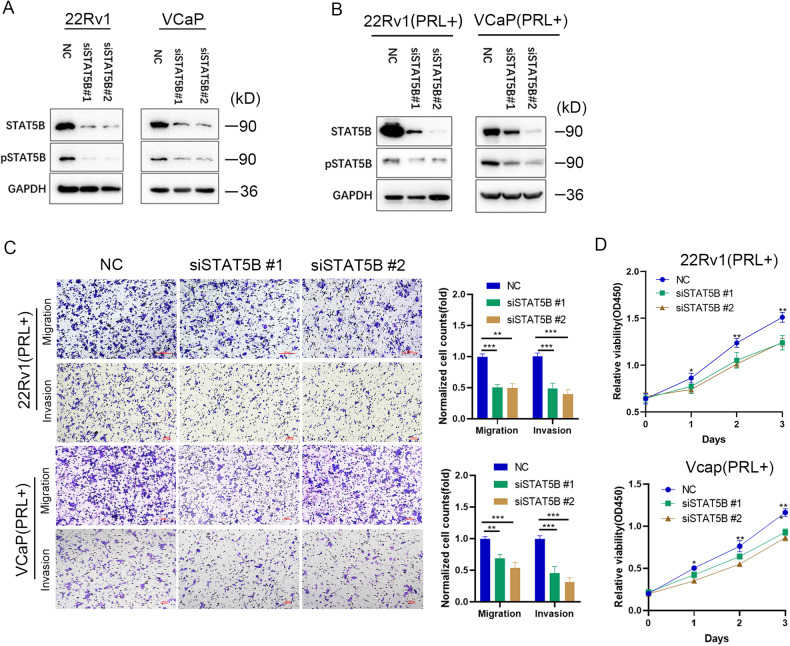
Effect of STAT5B knockdown on prostate cancer cells. **A** pSTAT5B levels after STAT5B knockdown in 22Rv1 and VCaP cells without PRL stimulation. **B** pSTAT5B levels after STAT5B knockdown in 22Rv1 and VCaP cells with PRL stimulation. **C** Influence of STAT5B knockdown on migration and invasion abilities of 22Rv1 and VCaP cells, and (**D**) influence of STAT5B knockdown on proliferation of 22Rv1 and VCaP cells.

To study the effect of STAT5B interference on PRL stimulation, we conducted STAT5B knock down and examined its significance in inducing STAT5B phosphorylation. The results demonstrated a substantial decrease in pSTAT5B levels following STAT5B knockdown (Fig. 3A). Furthermore, STAT5B knockdown significantly inhibited the PRL-induced phosphorylation of STAT5B (Fig. 3B). Transwell experiments indicated that STAT5B knockdown effectively reduced the migration and invasion capabilities of 22Rv1 and VCaP cells (Fig. 3C). The proliferation assay also revealed that STAT5B knockdown delayed the proliferation of 22Rv1 and VCaP cells (Fig. 3D). These findings suggest that reducing STAT5B phosphorylation can suppress the malignancy of PCa induced by PRL stimulation. Hence, STAT5B holds a potential therapeutic target for PCa.

### Identification of STAT5B target genes in prostate cancer

**Fig. 4:**
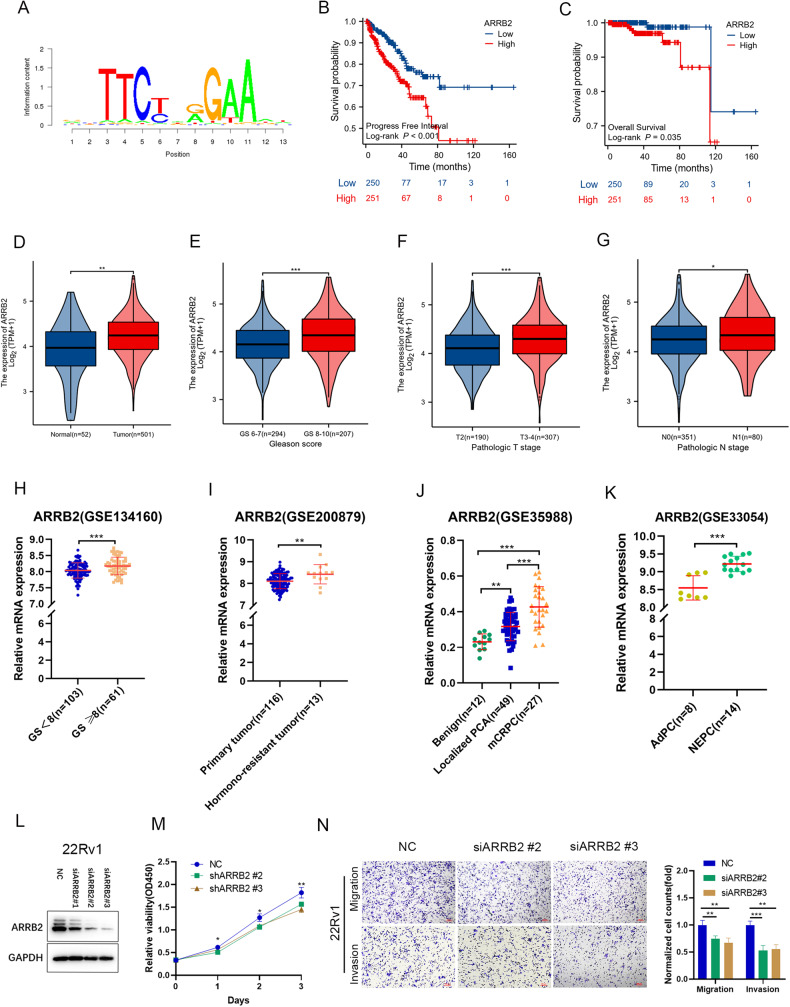
Potential target genes of STAT5B in prostate cancer. **A** Motif of STAT5B. **B** Influence of ARRB2 expression levels on the progression-free survival and (**C**) overall survival of patients in The Cancer Genome Atlas(TCGA) database. **D** Comparison of ARRB2 expression levels between normal and tumor tissues, and (**E**) comparison of ARRB2 expression levels between Gleason score 6-7 vs. 8–10, (**F**) T1-2 vs T3-4, (**G**) N0 vs N1. Expression levels of ARRB2 in Gene Expression Omnibus (GEO) database: (**H**) Gleason score (GS) < 8 vs. GS ≥ 8, (**I**) Primary tumor vs. hormone-resistant tumor. **J** Benign and localized prostate cancer vs. mCRPC, and (**K**) Adenocarcinoma prostate cancer (AdPC) vs. Neuroendocrine prostate cancer (NEPC). **L** ARRB2 knock down (siARRB2) in 22Rv1 cells. **M** Effect of siARRB2 on proliferation, (**N**) migration and invasion abilities of 22Rv1 cells.

STAT5B, as a transcription factor, regulates the expression of target genes [25]. We employed the Cistrome DB database to identify potential target genes, utilizing ChIP-seq data of STAT5B [26, 27] (Fig. 4A). A list of highly correlated target genes (Score ≥ 5.0) was found (Supplementary Table 2). Among these genes, *ARRB2* was a significantly poor prognostic marker for PFS (*HR* = 4.54, *P* = 0.035) and OS (*HR* = 2.01, *P* < 0.001) of PCa patients based on the TCGA-PRAD database (Fig. 4B, C). Furthermore, ARRB2 exhibited higher expression levels in tumor tissues compared to normal tissues (Fig. 4D**)**, and its expression increased with a higher Gleason score (Fig. 4E), advanced T stages (Fig. 4F), and N stages (Fig. 4G). Analysis of GEO databases also demonstrated elevated ARRB2 expression in tissues with higher Gleason scores (Fig. 4H).In addition, CRPC tissues exhibited significantly higher ARRB2 expression compared to primary tumor tissues (Fig. 4I, J), and ARRB2 was found to be more highly expressed in NEPC tissues compared to adenocarcinoma PCa tissues (Fig. 4K). To further investigate the role of ARRB2 in PCa, *ARRB2* knockdown were conducted in 22Rv1 cells (Fig. 4L), the results showed inhibition of proliferation, migration, and invasion ability of PCa (Fig. 4M, N). These results showed ARRB2 was a potential target gene of STAT5B, and higher ARRB2 expression indicated high aggression of PCa.

### STAT5B transcriptionally regulates the expression of ARRB2 in prostate cancer

**Fig. 5:**
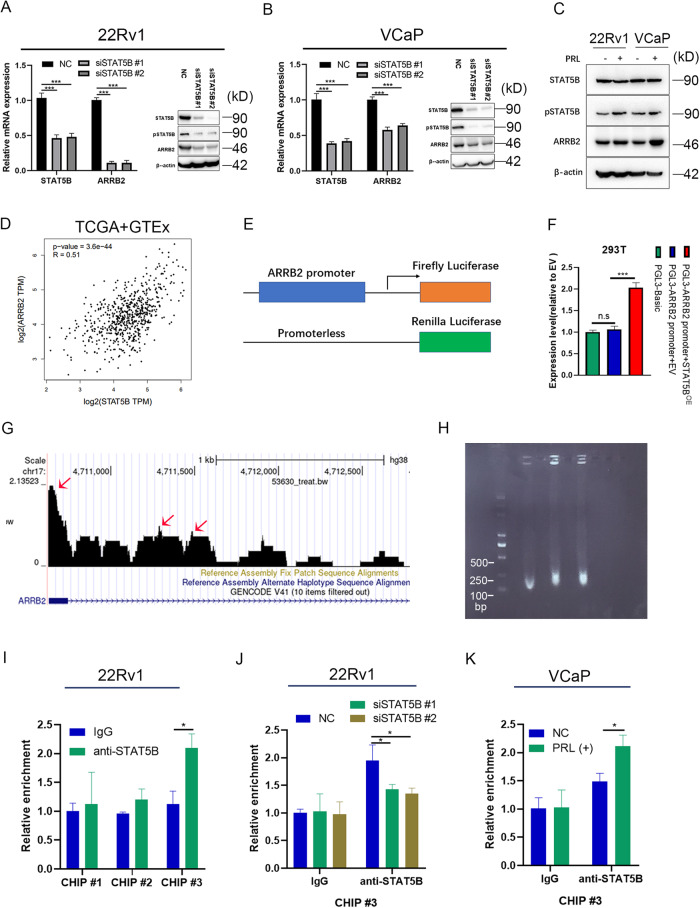
Transcriptional relation between STAT5B and ARRB2. **A** Influence of STATTB knockdown on the expression of ARRB2 in 22Rv1 and VCaP cells (**B**). **C** Effect of PRL stimulation on the expression of ARRB2 in 22Rv1 and VCaP cells. **D** Correlation of STAT5B and ARRB2 expression in The Cancer Genome Atlas (TCGA) and The Genotype-Tissue Expression (GTEx) database. **E** Structure of the constructs used in the luciferase reporter gene tests. **F** Fold change of ARRB2 promoter activity in 293 T cells with STAT5B overexpression and control group. **G** Potential binding sites of STAT5B on the ARRB2 promoter predicted in UCSC database. **H** Size of DNA fragments after ultrasonic fragmentation. **I** Enrichment of different primers in anti-STAT5B ChIP samples. **J** Enrichment of primer #3 in anti-STAT5B ChIP samples with STAT5B knockdown 22Rv1 cells, and (**K**) enrichment of primer #3 in anti-STAT5B ChIP samples with PRL stimulation in VCaP cells.

To further study the relation between STAT5B and ARRB2, STAT5B was knocked down in 22Rv1 and VCaP cells. The results demonstrated a decrease expression of *ARRB2* subsequent STAT5B knockdown (Fig. 5A, B). Additionally, PRL stimulation resulted in the upregulation of ARRB2 expression (Fig. 5C). Data from TCGA and GTEx database also showed a significantly positive correlation between STAT5B and ARRB2 expression levels (Fig. 5D). We then performed luciferase reporter gene assays to validate the transcriptional relationship between STAT5B and *ARRB2*, indicating a significant increase in *ARRB2* promoter activity after STAT5B overexpression (Fig. 5E, F). In the UCSC database, three potential binding sites of STAT5B were identified on the promoter region of ARRB2 (Fig. 5G). Subsequently, ChIP assay and qPCR were performed using anti-STAT5B with 200–250 bp DNA fragmentations (Fig. 5H), the results showed that STAT5B was enriched at the promoter region (primers ChIP #3) of ARRB2 in 22Rv1 cells (Fig. 5I). Furthermore, STAT5B knockdown resulted in reduced enrichment of the ARRR2 promoter (Fig. 5J), and PRL stimulation in VCaP cells showed an increased enrichment of the ARRR2 promoter (Fig. 5K). These findings support the transcriptional regulation relationship between STAT5B and *ARRB2*.

### Promotion of ERK1/2 phosphorylation through ARRB2 interaction in prostate cancer

**Fig. 6:**
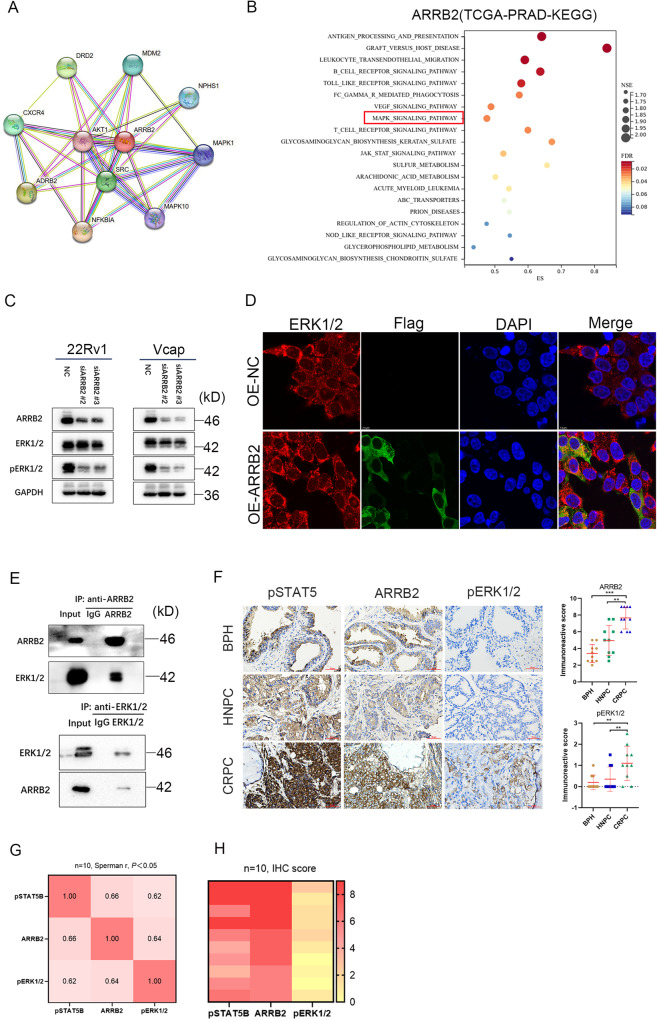
Relation between ARRB2 and MAPK signaling in prostate cancer. **A** Protein interaction networks of ARRB2 in String database. **B** Gene Set Enrichment Analysis (GSEA) of ARRB2 in TCGA-PRAD database. **C** Effect of ARRB2 knockdown (siARRB2) on phosphorylation of ERK1/2 (pERK1/2). **D** Immunofluorescence (IF) staining of ERK1/2 and Flag in 22Rv1 cells with ARRB2 overexpression (FLAG-tag). **E** Co-Immunoprecipitation test with anti-ARRB2 and anti-ERK1/2 in 22Rv1 cells. **F** Immunohistochemical (IHC) staining of pSTAT5B, ARRB2 and pERK1/2 in prostate tissues including castration resistant prostate cancer(CRPC), hormone naive prostate cancer (HNPC) and benign prostate hyperplasia (BPH), and the comparison of ARRB2 and pERK1/2 expression levels in different pathological tissues. **G** Correlation between the expression levels of pSTAT5B, ARRB2, and pERK1/2 in CRPC tumor tissues. **H** IHC score of pSTAT5B, ARRB2, and pERK1/2 in CRPC tumor tissues.

ARRB2 has been identified as a signaling scaffold for MAPK pathways and combining with ERK1/2 and promoting the phosphorylation of ERK1/2 (pERK1/2) [28, 29]. Protein interaction analysis using the STRING Database revealed that ARRB2 interacts with MAPK signaling components (Fig. 6A). GSEA analysis of the TCGA-PRAD database showed a higher enrichment of ARRB2 in the MAPK signaling pathway (Fig. 6B). We further investigated the relationship between ARRB2 and ERK1/2 in PCa cell lines. siARRB2 was used to silence ARRB2 expression, and this resulted in reduced levels of pERK1/2 without affecting total ERK1/2 levels in 22Rv1 and VCaP cell lines (Fig. 6C). Immunofluorescence (IF) staining demonstrated colocalization of ARRB2 (FLAG-tag) with ERK1/2 in the cytoplasm of 22Rv1 cells (Fig. 6D). Co-immunoprecipitation (Co-IP) method validated the interaction between ARRB2 and ERK1/2. Particularly, anti-ARRB2 or ERK1/2 pull-down assays enriched ERK1/2 or ARRB2, respectively (Fig. 6E). The IHC analysis of clinical samples revealed higher expression of ARRB2, and pERK1/2 in CRPC specimens compared to HNPC and BPH (Fig. 6F), and a positive correlation was found in the expression levels (IHC score) of pSTAT5B, ARRB2 and pERK1/2 (Fig. 6G, H). These results indicated that ARRB2 promotes the phosphorylation of ERK1/2, thereby activating the MAPK signaling.

### STAT5B knockdown inhibits MAPK signaling activation and prostate cancer growth in vivo

**Fig. 7:**
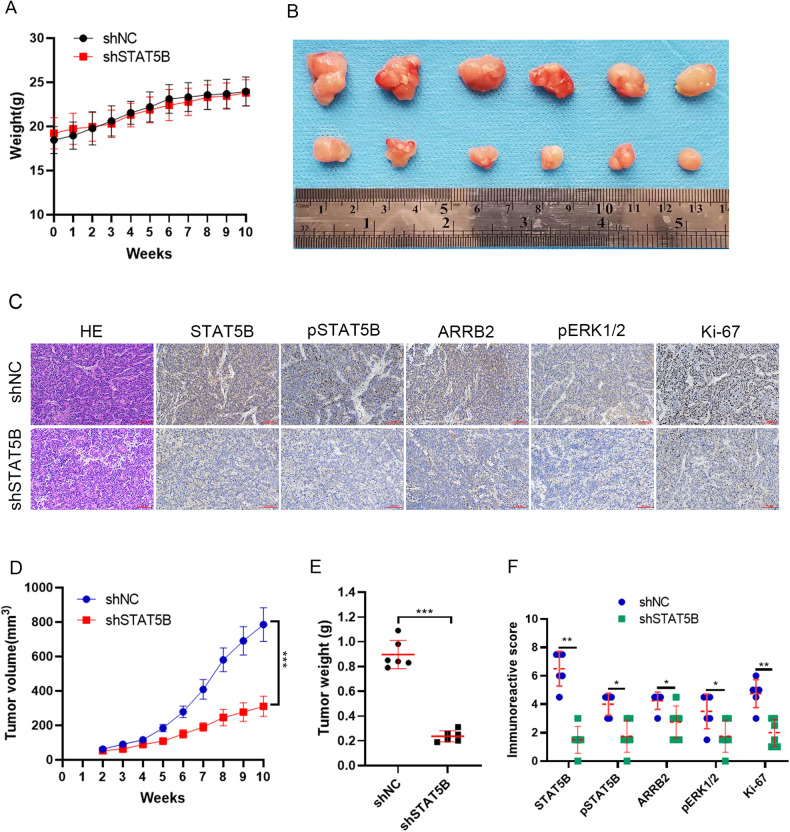
Influence of STAT5B knockdown on MAPK signaling and tumor growth in vivo*.* **A** Body weight of the nude mice after tumor implantation. **B** Image of the tumors after euthanization of the mice. **C** Immunohistochemical (IHC) staining of STAT5B, pSTAT5B, ARRB2, pERK1/2 and Ki-67 in tumor tissues. **D** Change in tumor volume between shSTAT5B and shNC group. **E** Comparison of tumor weight between shSTAT5B and shNC groups. **F** Comparisons of different molecules (STAT5B, pSTAT5B, ARRB2, pERK1/2 and Ki-67) between shSTAT5B and shNC groups.

To further investigate the role of STAT5B/ARRB2 and MAPK signaling in PCa, the 22Rv1 cells with stable STAT5B knockdown were implanted subcutaneously in nude mice. The tumor volume and weight of the nude mice were regularly monitored. The results showed that STAT5B knockdown significantly inhibited the growth of subcutaneous tumors without affecting the body weight of nude mice (Fig. 7A, B, D, E). The IHC was performed on tumor tissue from shSTAT5B and shNC groups, and the result indicated that the shSTAT5B downregulated the expression of ARRB2 and reduced the levels of pERK1/2 compared to the shNC group (Fig. 7C, F). Additionally, IHC staining of Ki67 showed significant inhibition of tumor proliferation in the shSTAT5B group (Fig. 7F). These results indicated that interferences with STAT5B diminish the expression of ARRB2, suppress MAPK signaling activation, and suppress PCa tumor growth in vivo.

## Discussion

Drug resistance poses a significant challenge in managing metastatic PCa. While many patients initially respond well to androgen deprivation therapy (ADT) or next-generation hormonal therapies (NGHT), they inevitably experience disease progression and drug resistance [30, 31]. PCa relies on androgen receptor (AR) signaling at the primary diagnosis [32–34]. However, as the development of drug resistance, other signaling pathways, independently or in collaboration with AR signaling, contribute to disease progression [35, 36]. Furthermore, the heterogeneity of the disease becomes increasingly evident [37–39]. Our previous study identified higher serum PRL levels as a poor prognostic factor for patients with mCRPC receiving abiraterone. We also observed higher local expression of PRL in CRPC specimens compared to BPH and HNPC [20]. Based on the previous study, this study investigated the heterogeneous expression of prolactin receptors (PRLR) in PCa. Stimulation with PRL led to the phosphorylation of STAT5B, which regulated the proliferation and invasion ability of PCa. Further investigations revealed that the *ARRB2* was a target gene of STAT5B. We observed increased expression of pSTAT5B, ARRB2, and pERK1/2 in CRPC specimens, and ARRB2 promoted the activation of the mitogen-activated protein kinase (MAPK) signaling pathway by interacting with ERK1/2 and promote the phosphorylation of ERK1/2.

Although the function of PRL and PRLR in PCa is not yet fully understood, previous studies have established STAT5 as a vital downstream mediator of PRLR [22, 40], and other downstream pathways, such as Src family kinases, phosphatidylinositol-3-phosphate kinase/Akt, TEC, NEK3 and EGFR/HER2, are also regulated by PRLR [41, 42]. Experimental studies have shown that phosphorylation of STAT5 regulates the biological functions of prostate and breast cancer cells [16, 17, 24], and the JAK2/STAT5 cascade appears to be the only pathway activated by PRL in prostate [43, 44]. However, a clinical trial of LFA102, a monoclonal antibody targeting PRLR, failed to demonstrate antitumor activity in prostate cancer and breast cancer [38]. In our study, we observed heterogeneous expression of PRLR in CRPC specimens and PCa cell lines, with STAT5B serving as a central molecule downstream of PRL in PCa. Related studies have shown that phosphorylation of STAT5 can be promoted by growth hormone receptors and interleukin-2 receptors, in addition to PRLR [45, 46], and the compensatory effects of other activators is unknow after block the function of PRLR in vivo. Moreover, our study showed that STAT5B knockdown significantly reduced pSTAT5B levels and inhibited the malignancy of PCa cells. These findings suggested that STAT5B could be a potential and effective target in managing PCa after resistance to hormonal therapy, and the role of other upstream pathways in STAT5 activation and progression of prostate cancer needs further exploration.

The pSTAT5B is an active form of STAT5, and the pSTAT5B can enter the nucleus and regulate target gene expression. Related study showed that a positive association of STAT5 activation with a high histologic grade of prostate cancer, and active STAT5 distinguished prostate cancer patients whose disease are likely to progress earlier [17, 47]. Experiment study also showed active STAT5 promotes metastatic behavior of human prostate cancer cells in vitro and in vivo [48]. However, the mechanism by which STAT5B promotes PCa progression is yet to be reported. Our study showed increased levels of pSTAT5B in CRPC tissues compared to HNPC and BPH tissues. Further investigation using public databases revealed *ARRB2* as a potential target gene of STAT5B. The TCGA and GEO databases showed *ARRB2* expression levels were correlated with tissue type, Gleason score, and N stage and were a poor prognostic factor in PCa. Additionally, ARRB2 knockdown significantly inhibited the malignant behaviors of PCa. We further validated that the STAT5B can bind to the promoter region of *ARRB2* and promote its expression by luciferase reporter gene tests and chromatin immunoprecipitation followed by quantitative PCR (ChIP-qPCR). Our data illustrates the regulation relation between STAT5B and ARRB2 in the progression of PCa, as STAT5B is an important transcription factor, further ChIP-seq data of STAT5B in PCa samples is needed to explore other target genes.

**Fig. 8:**
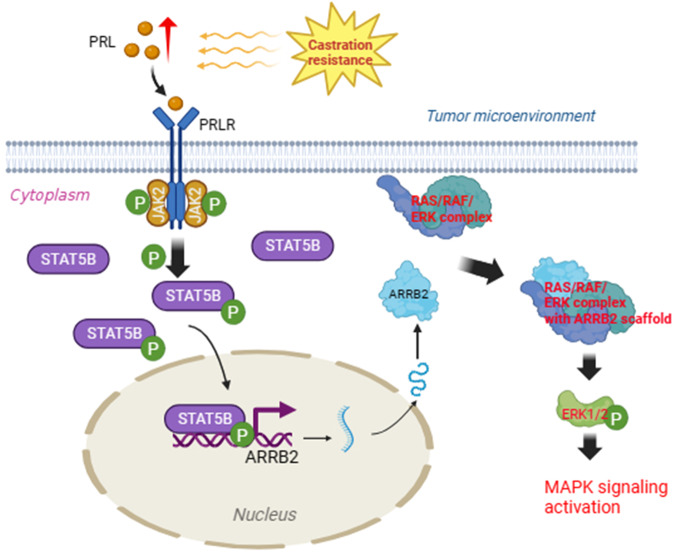
A working model based on the current study. After castration resistant progression of prostate cancer, rising PRL in tumor microenvironment promotes the phosphorylation of STAT5B, active STAT5B up-regulate the expression of ARRB2, and ARRB2 further enhance the activation of the MAPK signaling and promote the progression of prostate cancer by binding to ERK1/2 and enhance the phosphorylation of ERK1/2 (pERK1/2).

ARRB2, encoding beta-arrestin 2 proteins, is a multifunctional scaffolding protein in cytoplasm and regulates the activation of many signaling pathways [49, 50]. Related studies showed that beta-arrestin is a necessary component of Wnt/beta-catenin signaling [51], and beta-arrestin 2 acts as a signaling intermediate through a kinase/phosphatase scaffold and promote the activation of AKT signaling [52]. ARRB2 has also been shown to act as a signaling scaffold for MAPK pathways by interacting with ERK1/2 and promoting their phosphorylation [29, 49, 53]. ARRB2 has been reported to play important roles in the progression of ovarian cancer, renal cell carcinoma, and intestinal tumors [54–56]. Activation of MAPK signaling has been associated with drug resistance and progression of PCa [13, 57–59]. Our study showed that increased expression of ARRB2 and pERK1/2 in CRPC specimens, and a positive correlation was found between ARRB2 and pERK1/2. Furthermore, we demonstrated that the down-regulation of ARRB2 in 22Rv1 and VCaP cells decreased pERK1/2 levels, and co-immunoprecipitation confirmed the interaction between ARRB2 and ERK1/2. In vivo*,* experiments showed that STAT5B knockdown inhibited tumor growth by suppressing the expression of ARRB2 and phosphorylation of ERK1/2. These results indicate that STAT5B promotes PCa progression by transcriptionally regulating the expression of ARRB2 and activating MAPK signaling. In conclusion, our study revealed heterogeneous expression of PRLR in CRPC specimens, and we identified STAT5B as the cardinal subtype of STAT5 in PCa. PRL stimulation increased the pSTAT5B levels and enhanced the aggressiveness of PCa, which could be blocked by STAT5B knockdown. Mechanism study demonstrated that STAT5B transcriptionally regulated the expression of ARRB2, and ARRB2 activated the MAPK signaling pathway by combining with ERK1/2 and promoting ERK1/2 phosphorylation (Fig. 8). These findings suggest that STAT5B/ARRB2 pathway could be a potential therapeutic target for PCa.

## Material and methods

### Immunohistochemistry staining

Ten cases each of benign prostate hyperplasia (BPH), hormone-naive prostate cancer (HNPC), and castration-resistant prostate cancer (CRPC) were collected from Shanghai Tongji Hospital for this study. The study was approved by the ethical committee of the Shanghai Tongji Hospital (Approve Number: 2018–009). IHC analysis of PRLR, signal transducer and activator of transcription 5 A (STAT5A), signal transducer and activator of transcription 5B (STAT5B), phosphorylated STAT5B(pSTAT5B), Arrestin Beta 2 (ARRB2) and pERK1/2 was performed using the primary antibody listed in Supplementary Table 3. The procedures were performed as described previously [20]. Breast tissue was considered a positive control for PRLR, STAT5A, and STAT5B. Two independent pathologists, uninformed by the staining antibodies and clinical pathological variables, visually scored the paraffin sections. The scoring was based on the staining intensity, graded as 0 (negative), 1+ (weak), 2+ (moderate), or 3+ (strong), and the percentage of stained tumor cells, graded as <10% (0), 10%–25% (1+), 25%–50% (2+), or >50% (3+). The product of the two scores was used to compare the differences between the different tissue types, with a maximum assigned immunoreactive score of 9.

### Cell culture and transfection

The PCa cell lines used in this study, including LNCaP, C4–2, VCaP, 22Rv1, and PC3, were obtained from the American Type Culture Collection (ATCC). All cell lines were routinely tested for mycoplasma contamination. The LNCaP, C4–2, 22Rv1, and PC3 cell lines were cultured in RPMI1640 medium (SIGMA) supplemented with 10% fetal bovine serum (FBS, Biological Industries), VCaP was cultured in DMEM (GIBCO) with 10% FBS and 1% sodium pyruvate.

PRL stimulation experiments were performed as described in previous studies on breast cancer [60, 61]. Briefly, after overnight cell starvation, different concentrations of human recombinant PRL (CYT-267, ProSpec, Israel) were added to the culture medium. After 30 min of stimulation, cells were harvested to measure the level of pSTAT5B through western blotting. An appropriate concentration of PRL (maximum pSTAT5B level) was selected to stimulate the PCa cells for 72 h, and cells can be used for further experiments with a continuous PRL stimulation environment.

For transient transfection of small interfering RNA (siRNA) or overexpression plasmids, 293 T or PCa cells were plated at 70% confluency 24 h before transfection. The siRNA or overexpression plasmids were transfected using Lipo2000 transfection reagent (Invitrogen, Carlsbad, CA, USA) at a final concentration of 20 μM according to the manufacturer’s instructions; the detailed information of siRNAs and overexpression plasmid was listed in Supplementary Table 4 and Supplementary Fig. 1. After 48 h of transfection, cells were harvested at 48 h, and cell extracts were examined for the expression of the targeted protein expression using quantitative RT-PCR and western blot analysis—the siRNA showing that the most efficient knockdown of targeted genes was selected for further experiments.

To establish stable knockdown cell lines for STAT5B, shRNA was inserted into a pLKO One vector. The shRNA sequences for STAT5B knockdown were 5ʹ-GUGGCGAGAUCUUGAACAATT −3ʹ, 22Rv1 cells were transfected with packaging plasmids (psPAX2 and PMD.2 G) and shRNA plasmids using polyethyleneimine (Sigma-Aldrich, MO, US). After 48 h of transfection, the supernatant containing the virus was collected, filtered, and the infected cells were selected using 2 μg/mL puromycin, cells with stable passage were selected for further experiments.

### Tumorigenicity assays

Animal experiments were performed in accordance with a protocol approved by the Institutional Animal Care and Use Committee of Shanghai Tongji hospital. 5–7 weeks old male nude mice (Shanghai SLAC Laboratory Animal Company) were used for experiments. To study the function of PRL stimulation on tumorigenicity assays in vivo, human recombinant PRL was diluted to 0.8 μg/μl in sterilized saline, Alzet minipumps (Model 2006, ALZA Corporation, USA) containing sterilized saline or diluted human recombinant PRL were implanted subcutaneously on day 1, and the delivery rates was 3.6 μl /24 h. On day 4, 2.0 × 10^6^ 22Rv1 cells in combined with Matrigel (1:1, Corning, USA) were injected subcutaneously into the right flank of mice, the experiments process was referenced to Xu, X’s study [62]. To study the role of STAT5B knock down on tumorigenicity assays, mice were injected with 2.0 × 10^6^ 22Rv1 cells with STAT5B stable knock down or empty vector (control group) as described above. The tumor size was measured weekly with calipers. The mice were put to death 6–10 weeks later, and the tumors were retained for further experiments.

### Quantitative RT-PCR

The total RNA was extracted from cell samples using TRIzol (SIGMA), and 1 µg of total RNA was reversed transcribed to cDNA using the 5 × PrimeScript RT Master Mix (Takara) according to the manufacturer’s instructions. The synthesized cDNAs were then subjected to quantitative real-time PCR analysis using the 2 × ChamQ Universal SYBR qPCR Master Mix (Vazyme) according to the manufacturer’s instruction. Real-time PCR for each sample was performed in a 20 µl reactions system using the QuantStudio™ 5 Real-Time PCR System (Thermo Fisher Scientific). The primer sequences used for PCR amplification can be found in supplementary table 5.

### Western blot analysis

Total protein from tissue and cell lines was extracted by using RIPA lysis buffer (PC101, Shanghai Epizyme Biomedical Technology Co., Ltd), and the protein concentration was determined by using the BCA Protein Assay Kit (ZJ101L, Shanghai Epizyme Biomedical Technology Co., Ltd). Fifty micrograms of proteins were separated on 10% SDS-PAGE gels and transferred to a nitrocellulose membrane. The membranes were then blocked with 5% skim milk and incubated at 4 °C overnight with the primary antibodies. The primary antibodies and their dilution ratios are given in Supplementary Table 3. Subsequently, the membranes were incubated with the appropriate secondary antibodies (HRP-labeled Goat Anti-Human IgG or HRP-labeled mouse Anti-Human IgG, Beyotime Biotechnology, Shanghai, China) at normal temperature(20–25 °C) for 1 h. The protein bands were visualized using the FluorChem E chemiluminescent detection system (Protein Simple, Bio-Techne, USA).

### CCK8 and transwell assay

For the proliferation assay, cells were plated onto 96-well plates at a density of 3000 cells per well from each condition of the cell lines were plated onto 96-well plates and the plates were cultured with 100 µl of RPMI-1640 supplemented with 10% FBS (or DMEM + 10% FBS for VCaP cells). Cells were cultured and incubated for 0, 24, 48, and 72 h. Cell viability was assessed using a CCK-8 kit (Beyotime) according to the manufacturer’s instructions. The absorbance at 450 nm was measured using a microplate reader (Thermo Scientific).

Matrigel Matrix (Corning, #354248)-coated transwell dishes (aperture 0.8 μm) were used for the invasion assay. The Matrigel was diluted 10-fold in an FBS-containing medium, and 100 μl of the diluted Matrigel was added to each transwell dish. Cells with previous experimental treatment were resuspended in 1% FBS medium at a concentration of 1 × 10^5^ cells/200 µL. Then 200 µl of the cell suspension was added to each transwell dish, and 700 µL of complete medium was added to the basal side of the dishes. After 12 h (24 h for VCaP cells), the cells were fixed and stained with a 0.1% crystal violet fixative solution for 25 min. Cells were cultured in the transwell dishes without Matrigel Matrix for the migration assay. Invaded or migration cells on the underside of the membrane were counted in five medium-power fields using the Image J software (version 1.53t, National Institutes of Health, USA).

### Bioinformatic analysis

#### STAT5B target gene screen

The Cistrome DB (http://cistrome.org) contains a comprehensive collection of transcription factors (TFs) and histone ChIP-seq data, which can be utilized to predict the target genes regulated by specific TFs. The Species was set as homo sapiens, and STAT5B was selected as the transcription factor, highly correlated (Score≥5.0) targeted genes of STAT5B were predicted based on the studies conducted by Mitra et al. and Liao et al. [26, 27]. with high quality control (% Top 5k peaks phastcon conservation profiles ≥ 95%). Furthermore, the TCGA-PRAD database was employed to correlate the predicted targeted genes and the prognosis of PCa patients (http://gepia2.cancer-pku.cn/#index). Additionally, the relationship between the targeted genes and clinical features of PCa was investigated. The JASPAR database (https://jaspar.genereg.net/) was employed to predict the motif and DNA-binding domain of STAT5B on the promoter region [63]. Briefly, we chose vertebrata as the taxonomic group in JASPAR database, and profile of STAT5B(ID:MA1625.1) was selected for further analysis, 2000kb sequence on the front region of ARRB2 with FASTA-formatted (NC_000017.11:4710632-4712632) was extracted from NCBI database (https://www.ncbi.nlm.nih.gov/gene/), and the DNA-binding domains of STAT5B were predicted by the scan function of JASPAR database(supplementary table 2). Subsequently, primers for ChIP qPCR were designed and selected based on the DNA-binding domains of STAT5B, which can be found in Supplementary Table 6.

#### Public databases explore

The TCGA-PRAD database of 492 PCa samples was utilized to investigate the STAT5A and STAT5B expression levels in PCa tissues. Moreover, the relationship between ARRB2, and clinicopathological characteristics, such as normal vs. tumor tissue, GS (<8 vs. ≥8), T_1-2_ vs. T_3-4_, and N_0_ vs. N_1_, was analyzed based on TCGA-PRAD database. The clinical values of ARRB2 expression, and the GEO databases, including GSE134160, GSE200879, GSE35988, and GSE33054, were studied. Gene Set Enrichment Analysis (GSEA) was performed to explore the potential pathways associated with STAT5B and ARRB2 based on the TCGA-PRAD database. The interaction network between proteins was built in the String database (https://cn.string-db.org/).

### Luciferase reporter gene test

The ARRB2 promoter region (NC_000017.11:4710632-4712632) containing the binding site for STAT5B was cloned into pGL3 constructs driving the firefly luciferase reporter gene. Three types of cells were used to assess ARRB2 promoter activity levels: 293 T wild-type, standard control, and cells with STAT5B overexpressing. These cells were co-transfected with the ARRB2 promoter-driven firefly luciferase construct and the promoterless Renilla luciferase construct (supplementary fig. 2). The transfection was performed using Lipofectamine 3000 (Invitrogen, Carlsbad, CA, USA) according to the manufacturer’s instructions. Luciferase activity was evaluated 24 h later by Dual-Luciferase® Reporter Assay System (E1910, Promega, USA), and the Luciferase activity signals were detected by EnVision Nexus Multimode Microplate Reader (PerkinElmer, Massachusetts, USA). The results were normalized by calculating the ratio of firefly luciferase activity to Renilla luciferase activity and expressed as relative luminescence units.

### Chromatin immunoprecipitation assay

ChIP assays were performed using a ChIP Assay Kit (#P2078, Beyotime). Pretreatment for each sample involved lysing 1 × 10^7^ 22Rv1cells grown in 10-cm dishes. The ChIP reaction was performed using anti-STAT5 B (ab32364, Abcam) at a concentration of 4 µg per sample), and IgG (ab90285, Abcam) was set as a control, also at 4 μg per sample. The Nuclear extract preparation, immunoprecipitation, and DNA purification steps were performed according to the protocol provided by Beyotime. Briefly, Cell samples were cross-linked with 1% formaldehyde for 10 min at 37 °C and were stopped by 1.1 ml Glycine Solution (10X) for 5 min at room temperature. washed twice with cold PBS containing 1 mM PMSF (P001; NCM Biotech; Suzhou, China) and harvested in SDS Lysis buffer (containing 1 mM PMSF) from the ChIP Assay Kit. Then, the Covaris M220 (Massachusetts, USA) was used to sonicate the sample (100 W, 3-4 times, 10 s on/20 s off) at 4 °C. Then protein A + G Agarose/Salmon Sperm DNA were used to preclear the whole cell lysate for 30 min at 4 °C. After the 2 % input sample was extracted, the sample were divided equally and incubated with anti-STAT5B or control IgG antibody overnight. Then, Protein A + G Agarose/Salmon Sperm DNA were added for 1 h-incubation at 4 °C and washed sequentially with Low-Salt Immune Complex Wash Buffer, High-Salt Immune Complex Wash Buffer, LiCl Immune Complex Wash Buffer and TE Buffer (twice) for 3 min at 4 °C rotation. DNA-protein complexes were eluted with elution buffer (1% SDS and 0.1 M NaHCO3) and de-crosslinked by adding 5 M NaCl and heating for 4 h at 65 °C. Then, the proteins were digested with proteinase K for 1 h at 45 °C, and the DNA segments were further purified by a DNA Purification Kit (D0033; Beyotime). The 22Rv1 cells with STAT5B knockdown or control group were cultured in 10-cm dishes with 1 × 10^7^ cells for ChIP assay. VCaP cells were cultured with 40 ng/mL PRL stimulation or sterilized ultrapure water (control group) for 72 h and were further used for ChIP assay. Quantitative RT-PCR was performed as described using 1 µl of eluted chromatin, and primers (supplementary table 6) with effective enrichment was used for further experiments. The enrichment levels are presented as a percentage of the total input and normalized to IgG.

### Co-immunoprecipitation

22Rv1 cells were lysed using RIPA lysis buffer (PC101, Shanghai Epizyme Biomedical Technology) supplemented with a protease and phosphatase inhibitor cocktail (P1005, Shanghai Epizyme Biomedical Technology). The supernatants of the lysates were collected after centrifugation. These lysates were then incubated separately with ARRB2 (10171-1-AP, Proteintech) at a concentration of 2 µg/100 μl for each sample and ERK1/2 (ab184699, Abcam) at a concentration of 2 µg/100ul for each sample. The antibodies overnight at 4 °C with constant rotation. Subsequently, the antibodies in the lysates were precipitated with protein A/G magnetic beads (#88802, Thermo Scientific) and washed with PBS. The proteins bound to the beads were denatured using a 2 × loading buffer (#P0288, Beyotime); the resulting samples were then used for western blot analysis.

### Immunofluorescence

The 22Rv1 cells were transfected with an ARRB2 Overexpression plasmid containing 3xFlag (Supplementary Fig. 3). The cells were then cultured on a glass-bottom cell culture dish for two days. The cells were then fixed with 4% paraformaldehyde at room temperature (20–25 °C) for 20 min and washed thrice with PBS. Then the cells were permeabilized with 0.2% Triton X-100 for 20 min at 4 °C, followed by two additional washes with PBS, and block with 1%BSA for 30 min. The cells were incubated with antibodies against Flag-mouse and ERK1/2-rabbit overnight at 4 °C. After that; the cells were washed twice with PBS and incubated with fluorescent secondary antibodies (mouse-488 and rabbit-561, Invitrogen) at room temperature for 1 h. The nuclei were counterstained with DAPI for 10 min. The stained cells were determined using an SP8 LIGHTNING confocal microscope (Leica, Mannheim, Germany).

### Statistical analysis

Demographic and clinical characteristics were analyzed using descriptive statistics. Frequency distributions and percentages were used to summarize categorical variables. The differences in continuous variables among groups were compared using a t-test or Mann–Whitney U test. The independent sample Kruskal–Wallis test or general linear model univariate analysis (paired samples) was performed for variables with multiple groups. The data analyses were performed using IBM SPSS Statistics 22.0 for Windows (IBM Corp, Inc., New York, USA) and GraphPad Prism 8.0.2 for Windows (Graph Pad Software, Inc., California, USA). Data were collected from three independent experiments, and are expressed as the mean ± standard deviation (SD). Statistical significance was indicated by *, **, or *** representing *p* values less than 0.05, 0.01, or 0.001, respectively. The statistical significance was set at *P* < 0.05.

### Reporting summary

Further information on research design is available in the Nature Research Reporting Summary linked to this article.

### Supplementary information

Merged supplementary information file

Reporting Summary

## Data Availability

Data available on request from the authors
