# Supplementary material for: PRL-mediated STAT5B/ARRB2 pathway promotes the progression of prostate cancer through the activation of MAPK signaling
Source: Cell Death Dis. 2024 Feb 10;15(2):128. doi: 10.1038/s41419-023-06362-2 (PMC10858970; doi:10.1038/s41419-023-06362-2)
Supplement: Supplementary file 1 — Merged supplementary information file [file 41419_2023_6362_MOESM1_ESM.pdf]

Supplementary figure and table legends

Supplementary table 1. The clinical features of patients with prostate sample for immunohistochemistry staining

Supplementary table 2. The potential target genes of STAT5B predicted by the Cistrome DB

Supplementary table 3. The antibodies used for immunohistochemistry and western blot

Supplementary table 4. The siRNAs used in the experiments

Supplementary table 5. The primers used for real-time PCR analysis

Supplementary table 6. The primers for the promoter region of ARRB2 in STAT5B ChIP qPCR experiments

Supplementary figure 1. The structure of the overexpression plasmid of STAT5B

Supplementary figure 2. The structure of the overexpression plasmid of the ARRB2 promoter

Supplementary figure 3. The structure of the overexpression plasmid of ARRB2

Supplementary table 1. The clinical features of patients with prostate sample for immunohistochemistry staining

(1) Patients with metastatic castration resistant prostate cancer

Patient number	Age	GS	TNM	PSA (ng/ml)	PRL (ug/l)	PRL IHC-score
1	67	5+4	$T_4N_0M_1$	98.74	10.81	2
2	81	5+5	$T_3N_1M_1$	267	16.91	2
3	83	4+4	$T_4N_0M_1$	384.2	10.66	2.5
4	66	4+4	$T_3N_1M_1$	142.4	10.47	0
5	77	5+5	$T_4N_1M_1$	106	4.7	0
6	74	4+3	$T_4N_0M_1$	83.56	11.8	3
7	43	4+5	$T_3N_0M_1$	55.42	10.46	1
8	60	5+5	$T_4N_0M_1$	180.8	22.8	2.5
9	89	5+5	$T_3N_1M_1$	20.36	9.53	7.5
10	68	4+4	$T_3N_1M_1$	10.09	13.81	6

(2). Patients with hormone naive prostate cancer

Patient number	Age	GS	TNM	PSA (ng/ml)	PRL (ug/l)	PRL IHC-score
1	67	4+4	$T_2N_0M_1$	28.96	9.94	0
2	60	5+5	$T_4N_1M_1$	738.1	14.82	6
3	77	4+3	$T_3N_1M_1$	349	14.89	0
4	67	5+4	$T_4N_1M_1$	87.6	6.1	3
5	83	5+5	$T_3N_1M_1$	1222	9.05	0
6	86	4+3	$T_2N_0M_0$	10.8	6.33	0
7	74	4+5	$T_3N_0M_1$	44.5	8.97	0
8	74	3+3	$T_3N_1M_0$	17.6	5.24	0
9	78	5+4	$T_3N_0M_1$	259.0	3.39	0
10	66	4+5	$T_2N_0M_0$	26.4	8.67	0

(3). Patients with benign prostate hyperplasia

Patient number	Age	GS	TNM	PSA (ng/ml)	PRL (ug/l)	PRL IHC-score
1	50	/	/	6.7	8.21	0
2	68	/	/	7.83	NA	0
3	84	/	/	8.69	9.55	0
4	33	/	/	11.60	7.49	0
5	66	/	/	11.8	5.61	0
6	75	/	/	3.99	6.96	0
7	82	/	/	9.81	NA	0
8	38	/	/	6.58	NA	0
9	79	/	/	4.57	NA	0
10	68	/	/	6.69	7.28	0

Supplementary table 2. The potential target genes of STAT5B predicted by the Cistrome DB

Target gene	Score	Coordinate	DFS		OS	
			P	HR	*P*	HR
KDM6B	5.879	chr17:7842677-7854795	0.29	1.3	0.22	2.2
LOC105370399	5.793	chr14:22556952-22559556	NA	NA	NA	NA
IL13	5.586	chr5:132658172-132661109	0.055	1.5	0.61	1.5
CFLAR	5.19	chr2:201122476-201170755	0.5	1.2	0.38	1.8
PARK7	5.18	chr1:7961653-7985281	0.7	1.1	0.44	0.61
RAPGEF1	5.179	chr9:131576769-131668321	0.12	1.4	0.89	0.92
SLAMF1	5.081	chr1:160608110-160647020	0.32	1.2	0.93	1.1
ARRB2	5.074	chr17:4715182-4721499	0.001	2	0.018	5.7
GPR171	5.07	chr3:151197831-151203215	0.44	1.2	0.78	1.2
GPR68	5.057	chr14:91232531-91244409	0.001	2	0.59	1.4
DOCK10	5.037	chr2:224768252-224947064	0.028	1.6	0.36	1.8

Supplementary table 3. The antibodies used for immunohistochemistry and western blot

Antibodies	Dilution ratio of application		Manufacturers
	Immunohistochemistry	Western blot	
PRL	/	1:2000	Abcam (ab188229)
PRLR	1:800	1:1000	Invitrogen (1A2B1)
STAT5A	1:100	1:1000	CST (#25656)
STAT5B	1:500	1:5000	Abcam (ab178941)
pSTAT5B	1:100	1:1000	Abcam (ab32364)
ARRB2	1:50	1:1000	Proteintech (10171-1-AP)
ERK1/2	/	1:10000	Abcam (ab184699)
pERK1/2	1:400	1:1000	CST (#4370)

Supplementary table 4. The siRNAs used in the experiments

siRNA	Sense strand（5’ to 3’）	Antisense strand (5’ to 3’)
Negative control	UUCUCCGAACGUGUCACGUTT	ACGUGACACGUUCGGAGAATT
siSTAT5B #1	CCGCCAUAUAUUGUACAAUTT	AUUGUACAAUAUAUGGCGGTT
siSTAT5B #2	GUGGCGAGAUCUUGAACAATT	UUGUUCAAGAUCUCGCCACTT
siARRB2 #1	GUGCUAAAUCACUAGAAGA	UCUUCUAGUGAUUUAGCAC
siARRB2 #2	GUCAAGAAGAUCAAAGUCU	AGACUUUGAUCUUCUUGAC
siARRB2 #3	CAGAUGAUGACAUUGUGUU	AACACAAUGUCAUCAUCUG

Supplementary table 5. The primers used for real-time PCR analysis

Gene	sense	
GAPDH	Forword	TGTGGGCATCAATGGATTTGG
	Reverse	ACACCATGTATTCCGGGTCAAT
PRL	Forword	ACAAGGAGCAAGCCCAACAGATG
	Reverse	GTGGATTCGGCACTTCAGGAG
PRLR	Forword	TGACTTACCACAGGGAAGGAGAGAC
	Reverse	CATACAGGAGCGTGAACCAACAGATG
STAT5A	Forword	CGACGGGACCTTCTTGTTG
	Reverse	GTTCCGGGGAGTCAAACTTCC
STAT5B	Forword	GAACACCCGCAATGATTACAGT
	Reverse	ACGGTCTGACCTCTTAATTCGT
ARRB2	Forword	TCCATGCTCCGTCACACTG
	Reverse	ACAGAAGGCTCGAATCTCAAAG

Supplementary table 6. The primers for the promoter region of ARRB2 in STAT5B CHIP QPCR experiments

Primers		Senses
ARRB2-CHIP #1	Sense primer	TCCTTCAGAAAGACTGTCCTCATC
	Anti-sense primer	GGGTTCCCTTTTCCAATGTC
ARRB2-CHIP #2	Sense primer	GTGCAATCGAGGCTCTTTCC
	Anti-sense primer	TTTTGCAGAGCAAGCCAGAG
ARRB2-CHIP #3	Sense primer	GGGACCAGGTAAGGGAGGTG
	Anti-sense primer	CACGGGAGAGCCCAGTAGTC

Supplementary figure 1

The structure of the overexpression plasmid of STAT5B

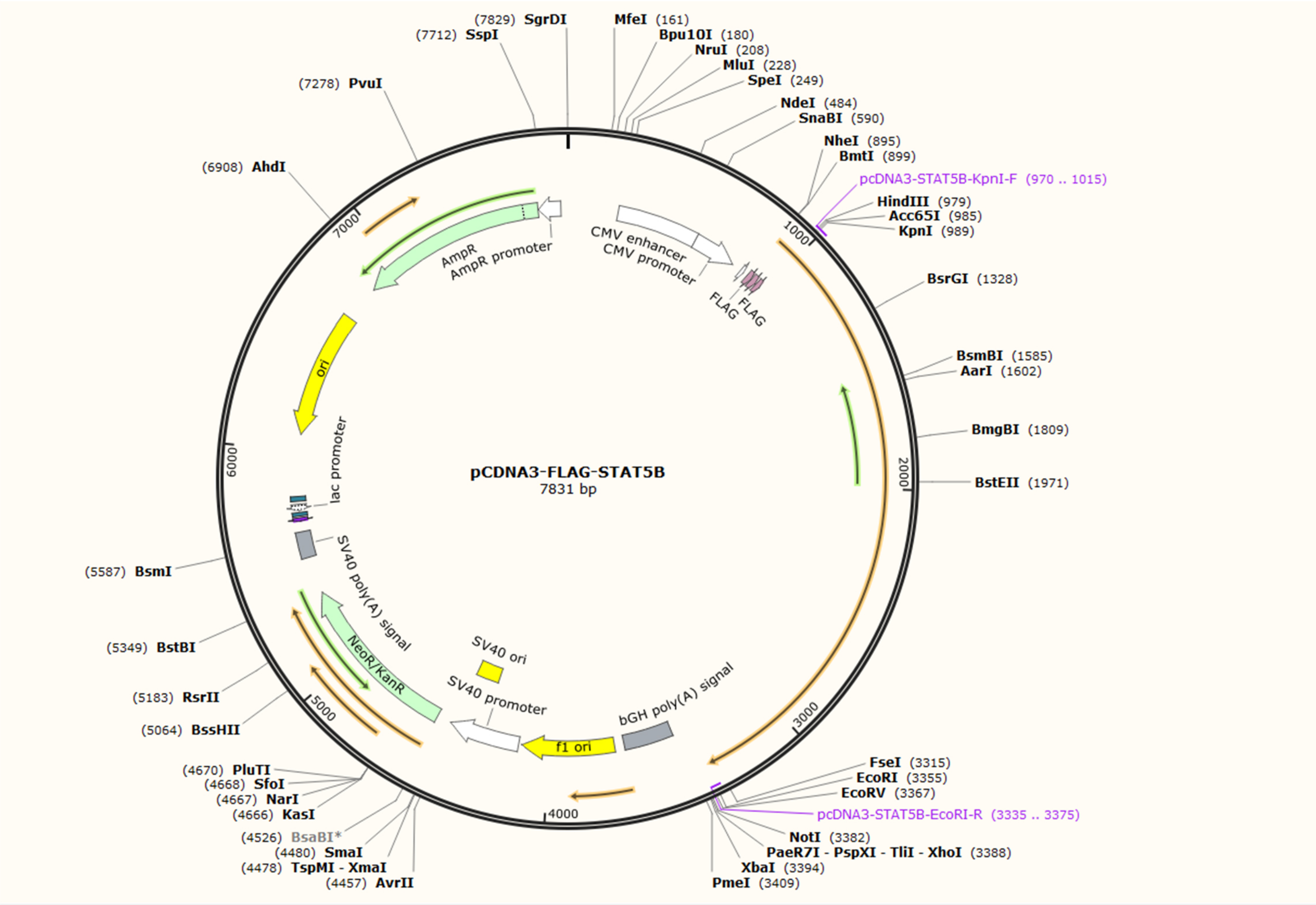

Supplementary figure 2

The structure of the overexpression plasmid of the ARRB2 promoter

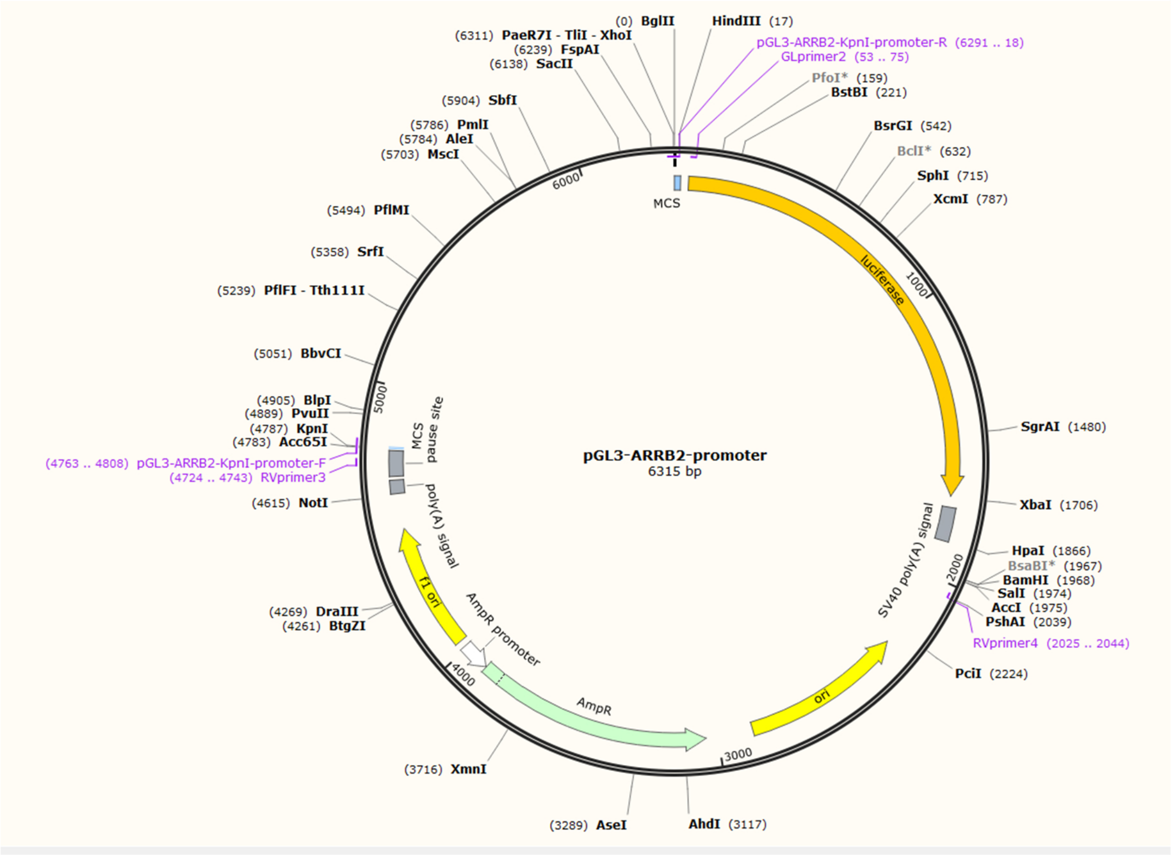

Supplementary figure 3

The structure of the overexpression plasmid of ARRB2

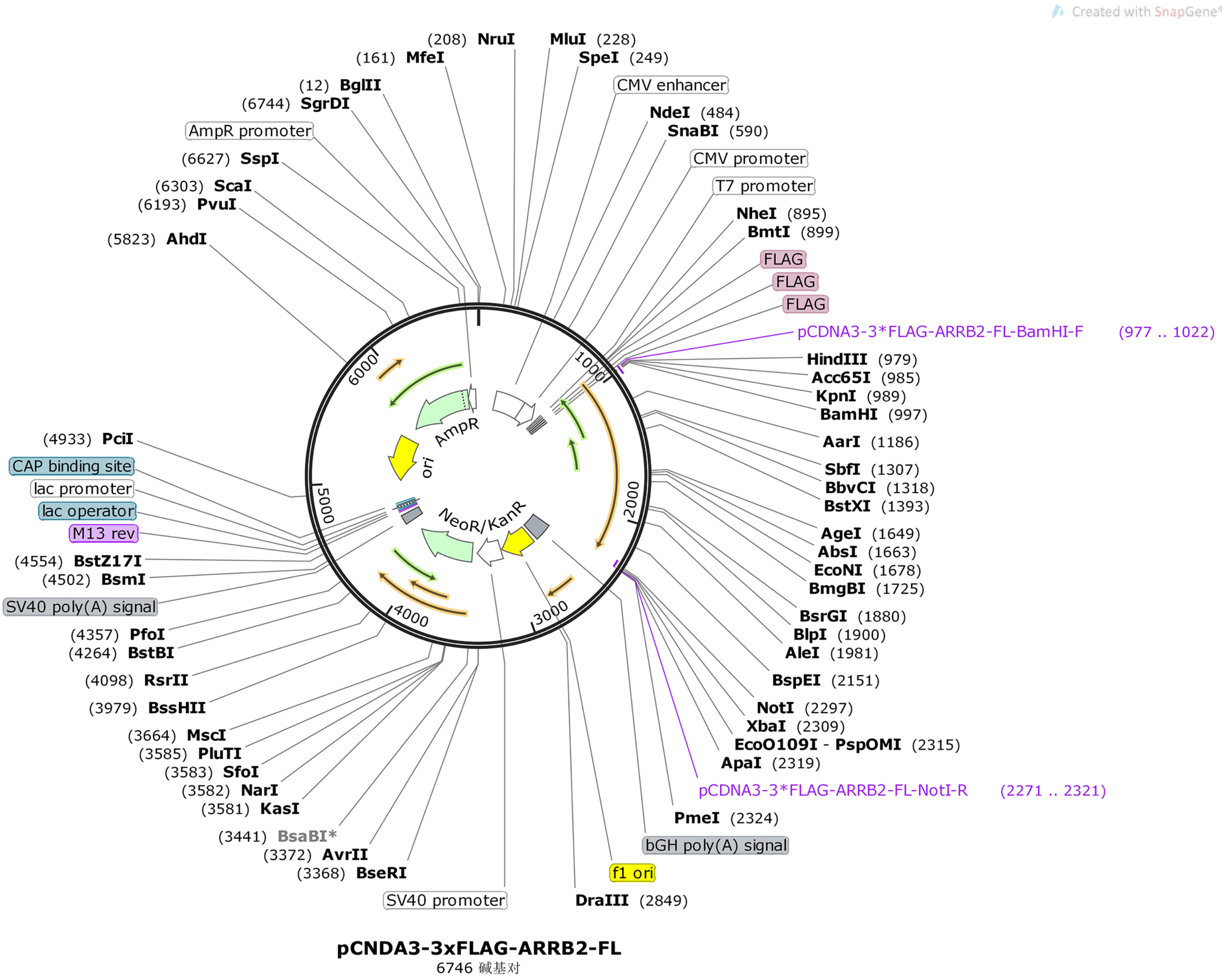

Original data Figure 1

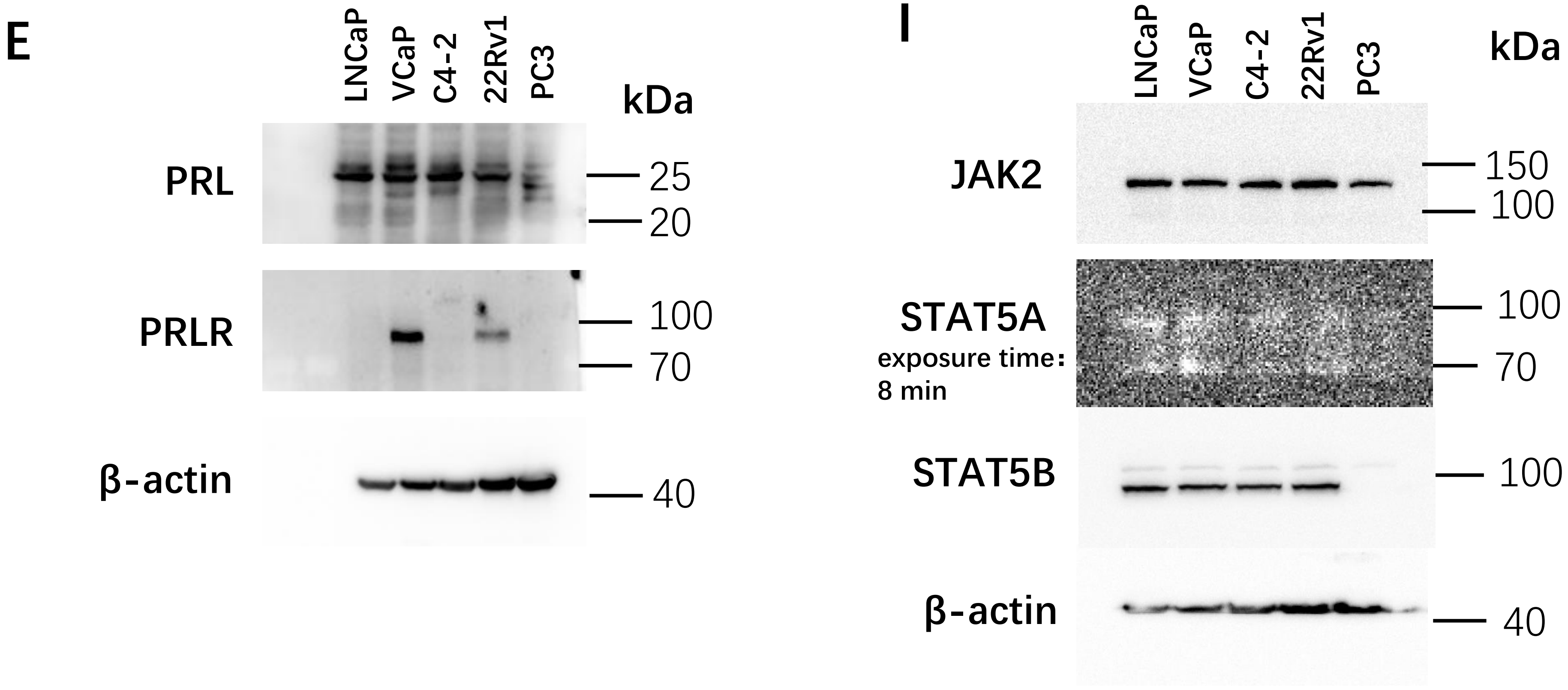

Original data Figure 2A

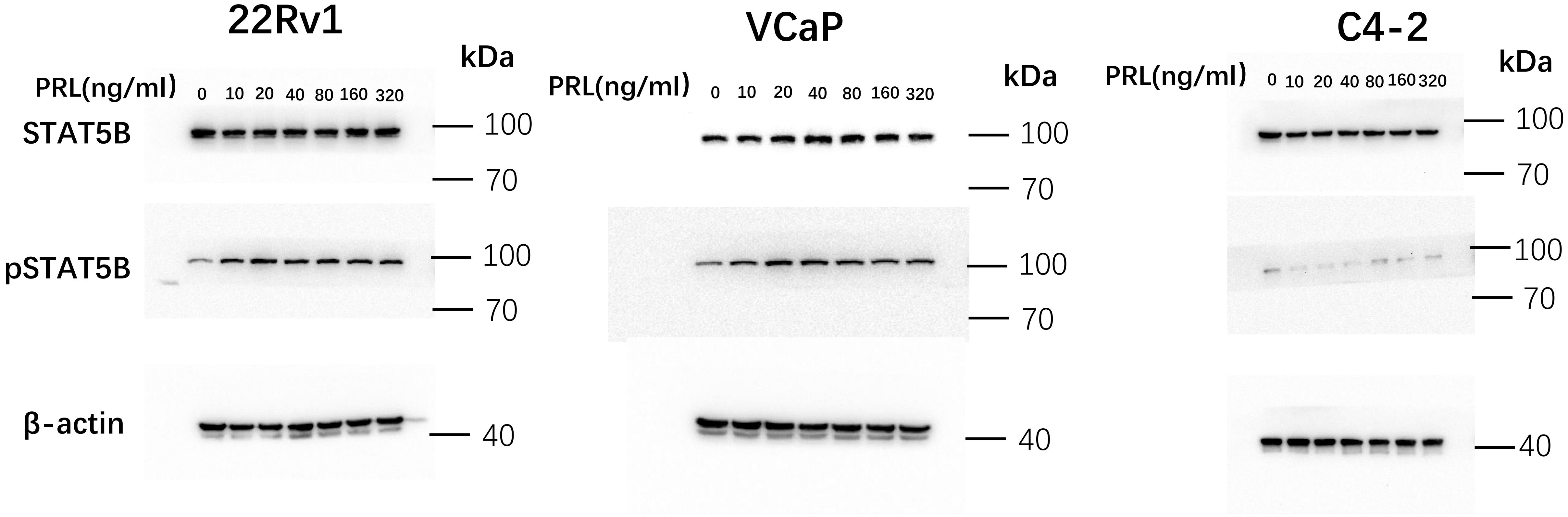

Original data Figure 2B

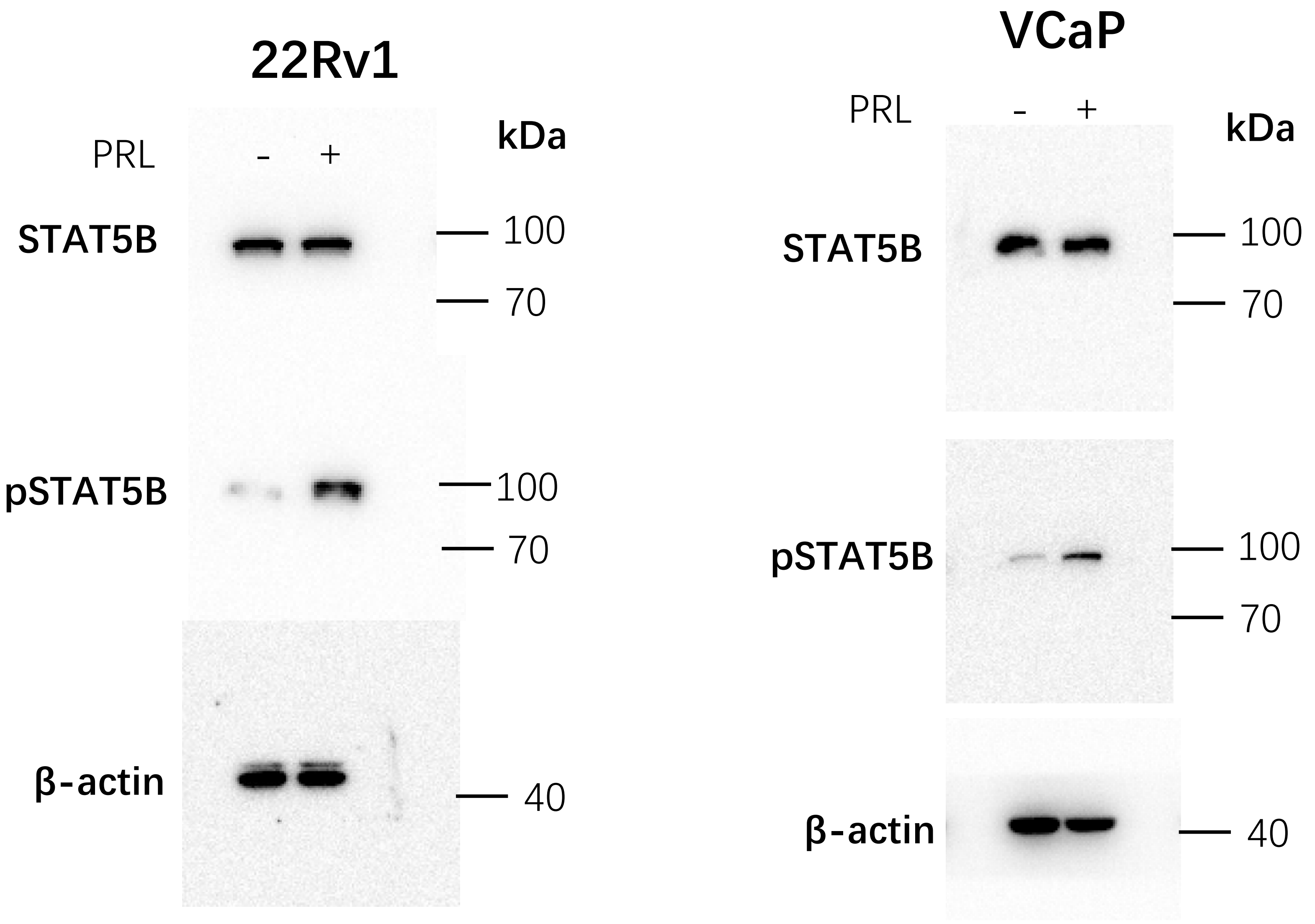

Original data Figure 3A

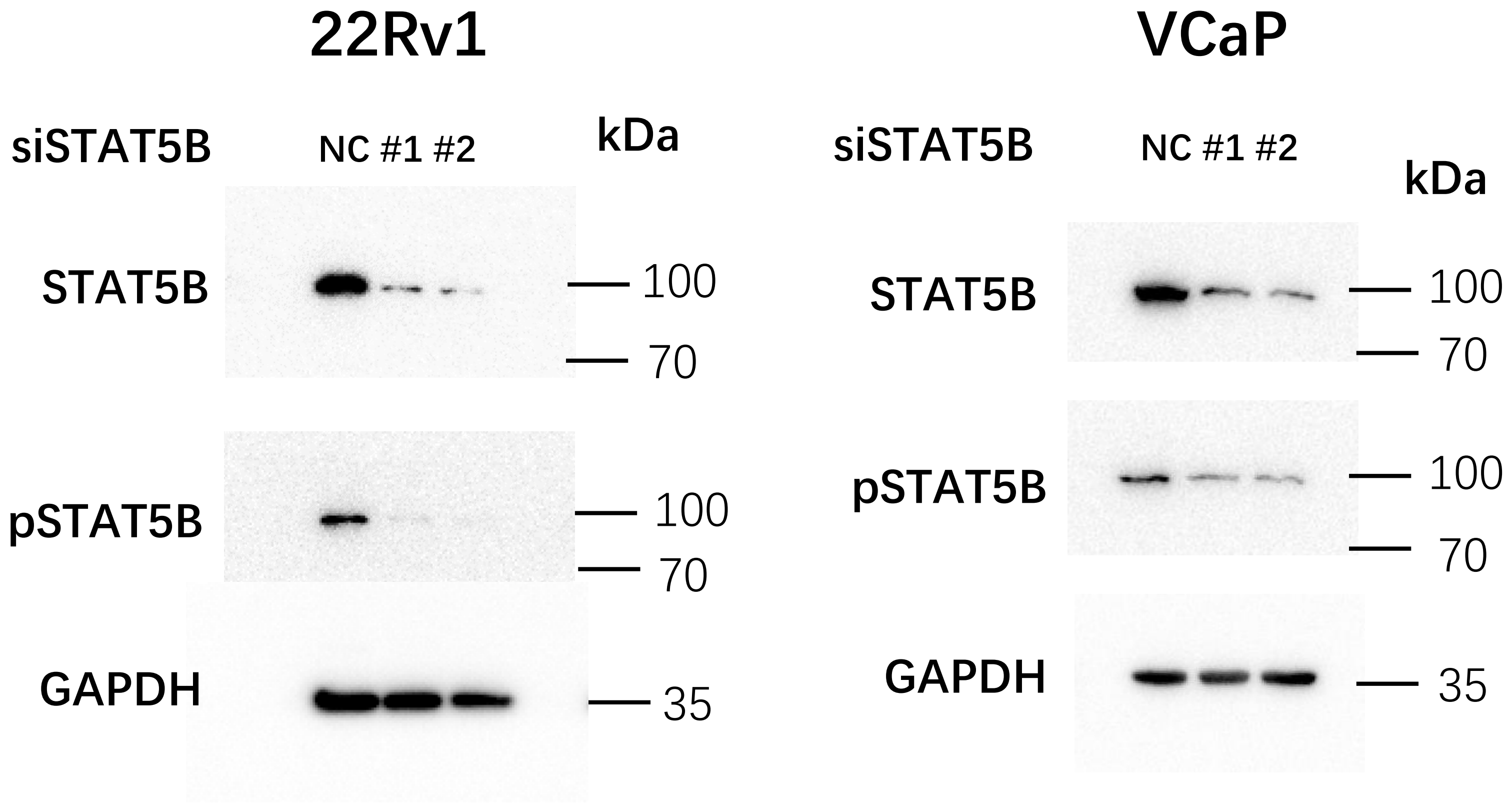

Original data Figure 3B

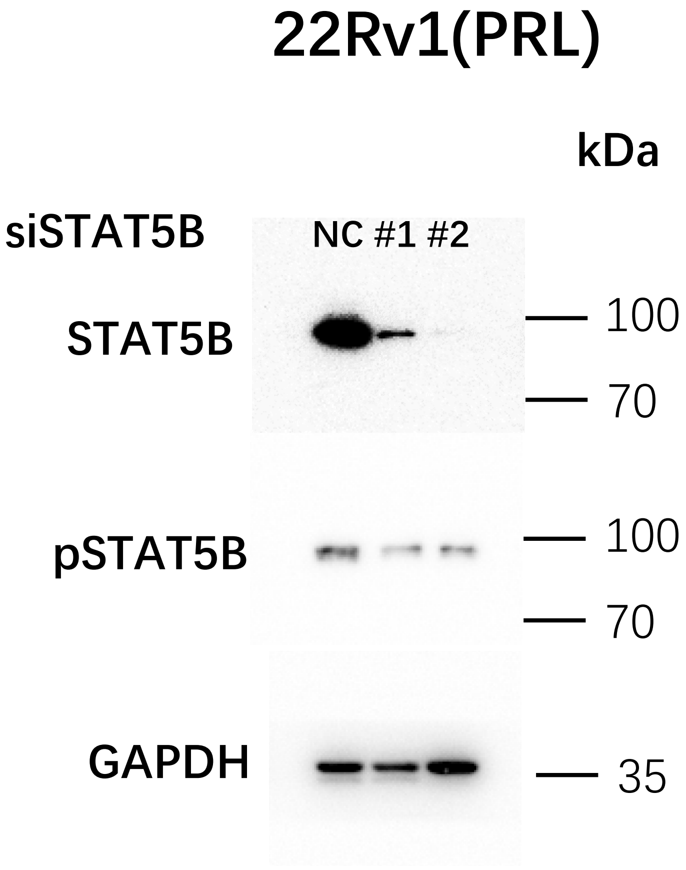

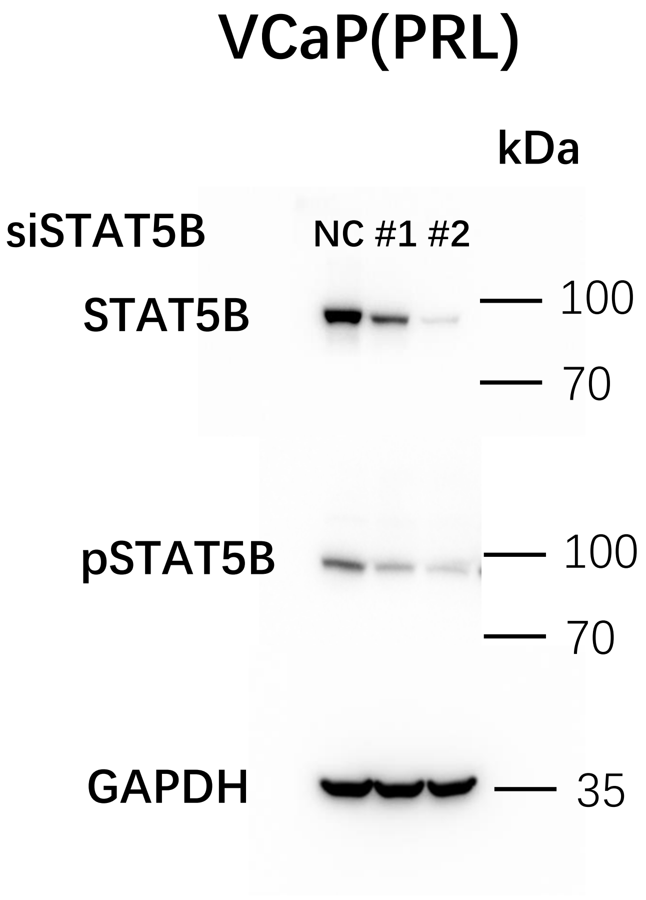

Original data Figure 4L

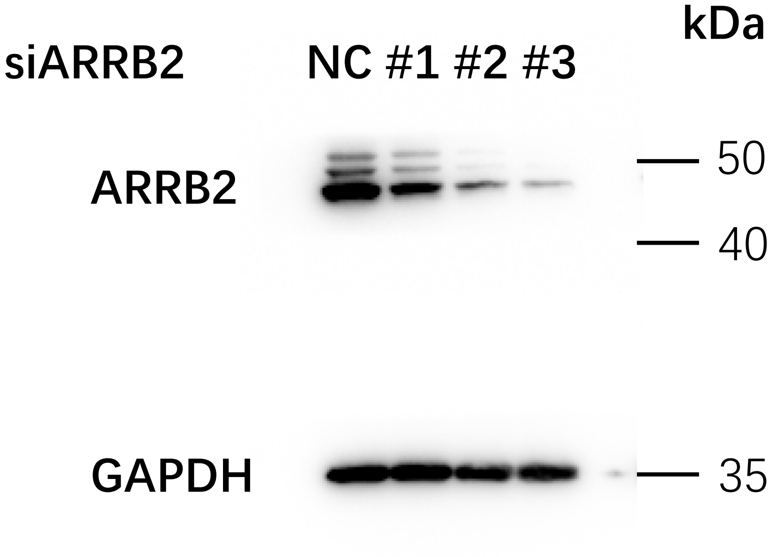

Original data Figure 5

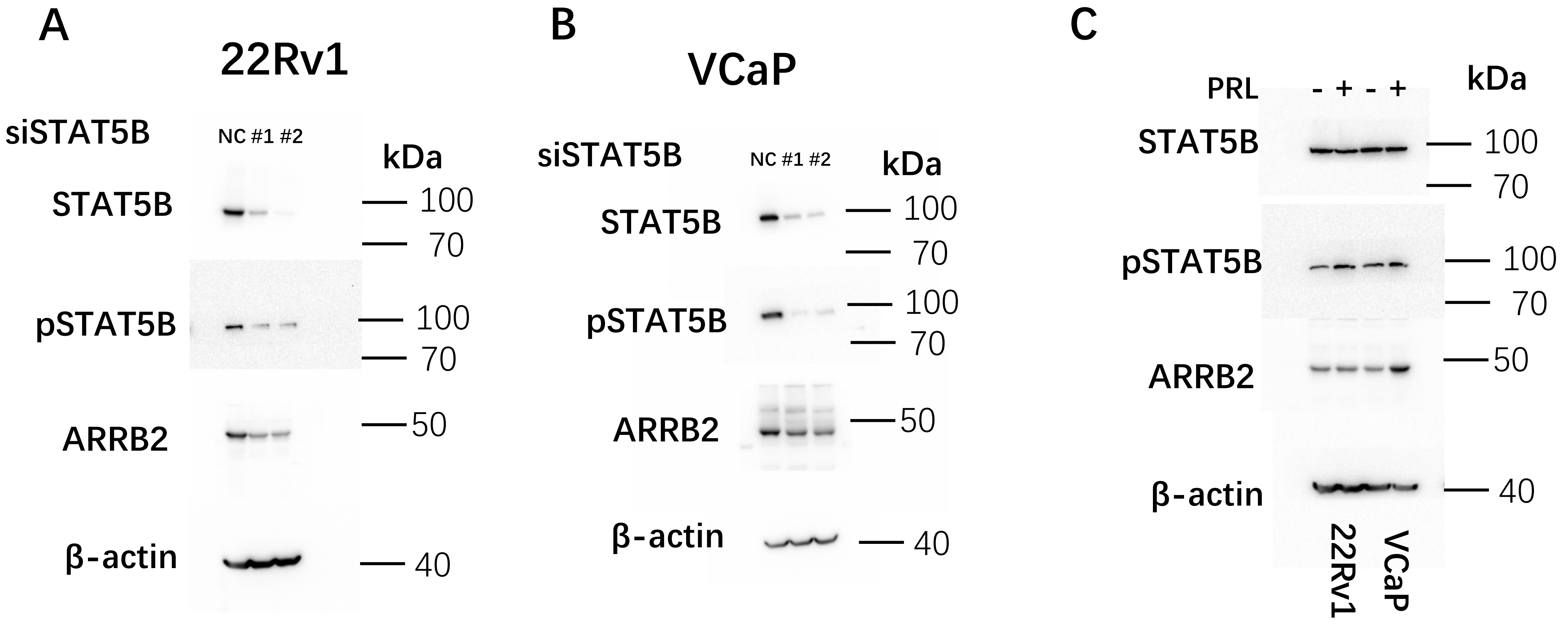

Original data Figure 6C

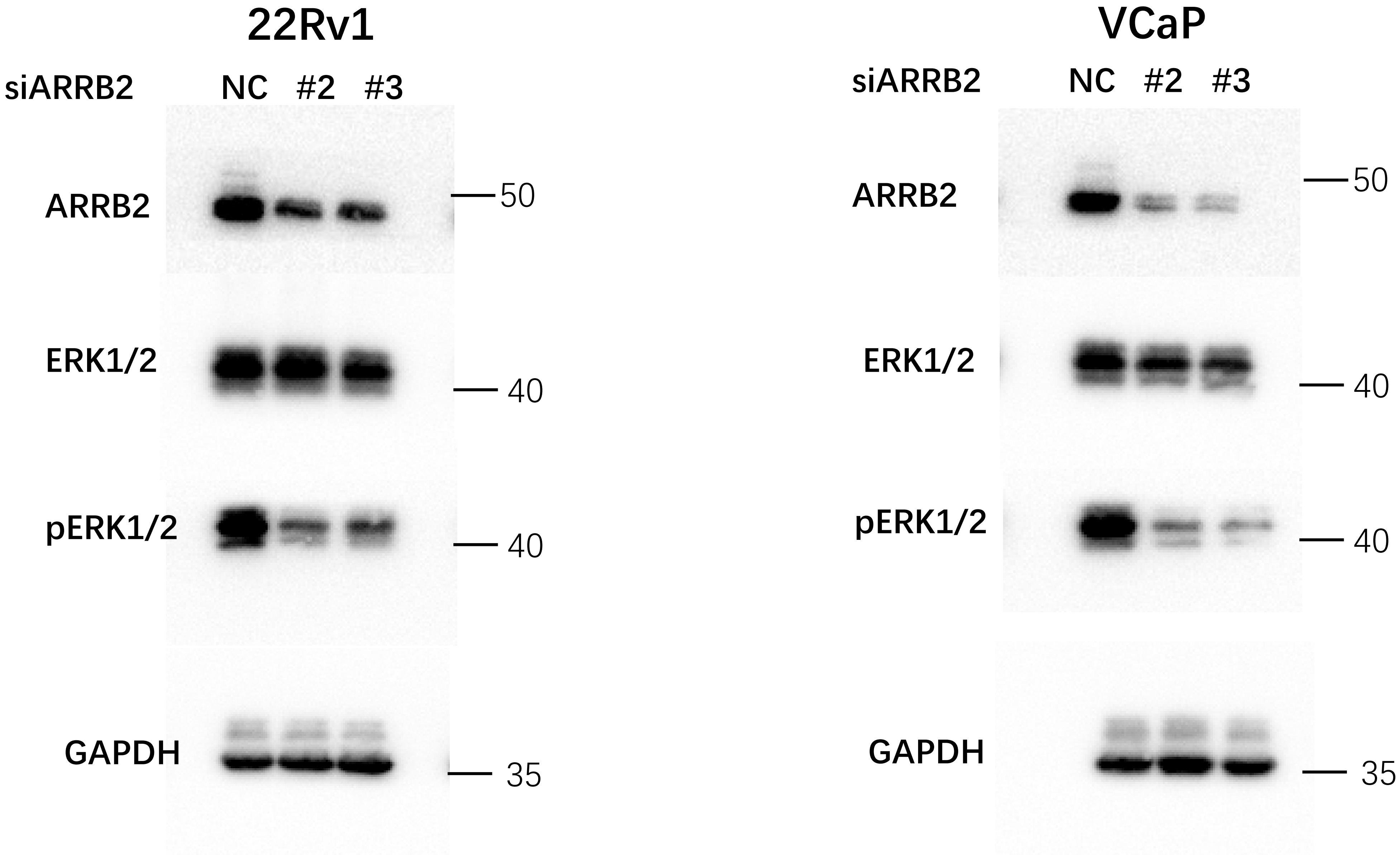

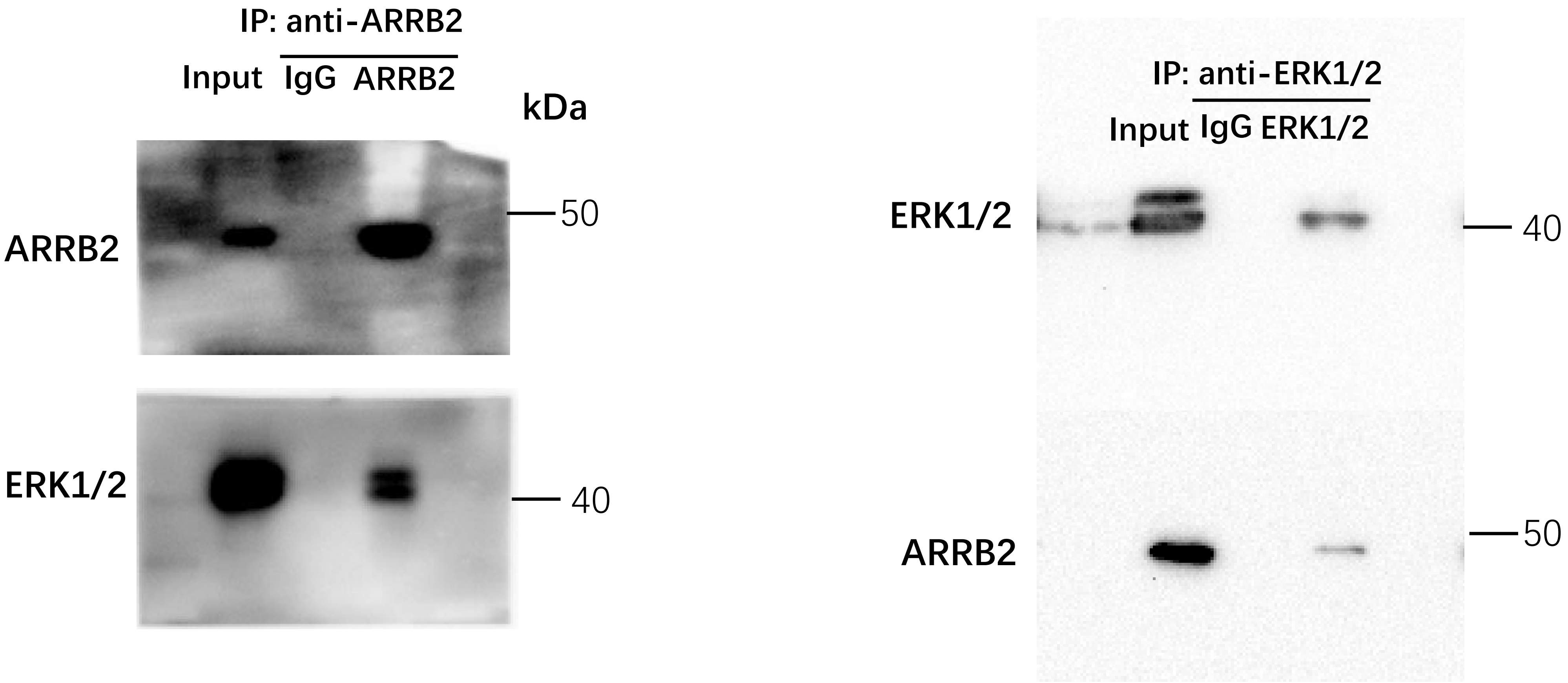
IP: anti-ARRB2
Input
IgG
ARRB2
kDa
ARRB2
50
ERK1/2
40
IP: anti-ERK1/2
Input
IgG
ERK1/2
ERK1/2
40
ARRB2
50
